# A multi-technique analytical approach to sourcing Scandinavian flint: Provenance of ballast flint from the shipwreck “Leirvigen 1”, Norway

**DOI:** 10.1371/journal.pone.0200647

**Published:** 2018-08-08

**Authors:** Michael Brandl, Maria M. Martinez, Christoph Hauzenberger, Peter Filzmoser, Pål Nymoen, Natascha Mehler

**Affiliations:** 1 Austrian Academy of Sciences, OREA-Institute, Vienna, Austria; 2 The University of Texas at Austin, Department of Anthropology, Austin, Texas, United States of America; 3 University of Graz, Institute of Earth Sciences, NAWI Graz Geocenter, Graz, Austria; 4 Vienna University of Technology, Institute of Statistics and Mathematical Methods in Economics, Vienna, Austria; 5 Norwegian Maritime Museum, Oslo, Norway; 6 University of Vienna, Institute for Prehistory and Historical Archaeology, Vienna, Austria; 7 Centre for Baltic and Scandinavian Archaeology, Schleswig, Germany; 8 Institute of Nordic Studies, University of the Highlands and Islands, Kirkwall, Orkney; University at Buffalo - The State University of New York, UNITED STATES

## Abstract

Although Scandinavian flint is one of the most important materials used for prehistoric stone tool production in Northern and Central Europe, a conclusive method for securely differentiating between flint sources, geologically bound to northern European chalk formations, has never been achieved. The main problems with traditional approaches concern the oftentimes high similarities of SiO_2_ raw materials (i.e. chert and flint) on different scales due to similar genetic conditions and higher intra- than inter-source variation. Conventional chert and flint provenance studies chiefly concentrate on visual, petrographic or geochemical investigations. Hence, attempts to generate characteristic fingerprints of particular chert raw materials were in most cases unsatisfying. Here we show that the Multi Layered Chert Sourcing Approach (MLA) achieves a clear differentiation between primary sources of Scandinavian flint. The MLA combines visual comparative studies, stereo-microscopic analyses of microfossil inclusions, geochemical trace element analyses applying LA-ICP-MS (Laser Ablation Inductively Coupled Plasma Mass Spectrometry) and statistical analyses through CODA (Compositional Data Analysis). For archaeologists, provenance studies are the gateway to advance interpretations of economic behavior expressed in resource management strategies entailing the procurement, use and distribution of lithic raw materials. We demonstrate the relevance of our results for archaeological materials in a case study in which we were able to differentiate between Scandinavian flint sources and establish the provenance of historic ballast flint from a shipwreck found near Kristiansand close to the shore of southern Norway from a beach source in Northern Jutland, the Vigsø Bay.

## Introduction

Scandinavian flint *sensu* Högberg and Olausson [1] can be regarded as one of the most important materials for prehistoric stone tool production in northwestern and parts of Central Europe. Previous studies have produced promising results for differentiating between several varieties of Scandinavian flint [2–5]; however, a comprehensive systematic study concerning this issue has not yet been presented. The current study focuses on the systematic characterization and differentiation of Scandinavian flint through the application of the Multi Layered Chert Sourcing Approach (MLA) [6]. This method combines macroscopic (visual), stereomicroscopic and geochemical analyses applying Laser Ablation Inductively Coupled Plasma Mass Spectrometry (LA-ICP-MS) for trace element detection. Multivariate geochemical data are subsequently evaluated by compositional data analysis (CODA) to achieve optimal separation between distinct geological source locations and best group assignment of individual samples to defined sources. The measured geochemical values, typically indicated in parts per unit, percentages, ppm, ppb, etc., have to be considered as raw compositional data (or compositions). Compositions represent “quantitative descriptions of the parts of some whole” [7], thus only conveying relative information. Their sample space is the so-called D-part simplex, also known as Aitchison simplex, for which standard statistical methods are not designed. Thus, compositional data need to be transformed into Euclidean geometry in which statistical methods can operate [7]. This can be best achieved by isometric logratio (ilr) transformation, which preserves all metric properties of compositions [8]. After transformation, discriminant analysis (DA) is applied. Optimal group separation of so-called training data derived from known geological sources can be achieved using Fisher’s linear discriminant analysis (LDA) [9]. The resulting discriminant rules are used for assigning the test data (i.e. the archaeological specimens) to these predefined groups (i.e. training data) [10].

Flint was an especially coveted raw material due to its exceptional flaking properties and could be procured from three distinct source contexts: From primary deposits by digging through the soft chalk layers which contain flint seams involving partly extensive mining operations, from secondary and sub-primary sources at beach shores around the Baltic and North Sea, and thirdly from glacial deposits extending as far south as Central Germany, Northern Bohemia and Moravia, and southwestern Poland. Thus, flint occurs abundantly in prehistoric Central and Northern European lithic assemblages.

The earliest evidence for the use of Scandinavian flint for chipped stone tool production is known from central Germany (Schladebach/Wallendorf) and dates to the Holstein interglacial (MIS 11, 340,000 to 325,000 BP) [11]. This material derived from erratic contexts. Due to its abundance and accessibility, erratic flint became of increasing importance during the Middle and Upper Paleolithic in Central and parts of Eastern Europe, e.g. south-central Germany and Poland, Hungary, Austria, Moravia and Bohemia [12]. The extensive use of erratic flint continued throughout the Mesolithic [13] and Neolithic periods in parts of Central and Eastern Europe [14–17].

In Scandinavia, hunter-gatherer groups arrived during the Late Upper Paleolithic c. 11,700 years ago [18, 19], and made extensive use of the rich flint resources they encountered. Characteristic small backed flint blades, so called “Federmesser”, became eponymous for a variety of North European Late Paleolithic groups [20, 21]. At the end of the fifth millennium BC, Neolithic populations arrived in Scandinavia [22]. From the fourth millennium on, regular flint mining from both, secondary and primary deposits, commenced in Denmark and Sweden [23, 24]. The most distinguished artifacts made from Scandinavian flint are daggers, which were widely circulated between the third and early second millennia BC over different parts of Europe [24–26]. The use of Scandinavian flint continued after the Bronze Age [27], and gained some economic importance for gunflint production at the end of the 18th century [28]. However, it never reached the industrial importance of the large British and French sources.

The present study demonstrates how the MLA achieves a discrimination of flint for archaeological sourcing studies—with Scandinavian flint used as ship ballast in historic times as case study to test the archaeological relevance of our results. This project was initiated within the framework of the interdisciplinary research project “Harbours of the North Atlantic c. AD 800–1300 (HaNoA)”. One component of the project is concerned with ship ballast as archaeological material culture, including the scientific study of lithic materials from submarine ballast sites. Owing to its abundance in the study area, flint plays an important role in this respect [29–31]. Scandinavian flint is bound to Cretaceous formations in Northern Europe and occurs, e.g., in Northern Germany, Denmark, Sweden and on the British Isles, directly coinciding with the HaNoA-project study area. Many primary flint deposits are situated at the coast, and weathered flint nodules could easily be collected from beach shores. For the archaeological case study, we investigated flint bearing limestone boulders used as ship ballast recovered from the post-medieval Leirvigen 1 shipwreck found off the southern shore of Norway, close to the town of Kristiansand.

Based on the nature of the ballast rocks it was apparent that they were acquired from a secondary beach source. In order to trace the original formation environment of the ballast material, clusters of primary flint sources were established. Through the investigation of secondary materials from beach deposits, we tested possibilities to assign such materials to an identified source area.

Our initial analytical step was to macroscopically and microscopically investigate already collected geological flint samples from potential source areas of the archaeological material, curated at the lithothec of the University of Vienna (Vienna Lithothek, VLI). This included materials from Great Britain, Denmark, south Sweden, Northern Germany, Lithuania and Belarus. Since only material from the Baltic area corresponded to the Leirvigen ship ballast, geological surveys were conducted at primary and secondary deposits in Denmark, southern Sweden and Northern Germany. The nature of primary and secondary deposits recorded during our geo-prospections revealed that the only potential sources of the ballast flint material were located either in Denmark or in Northern Germany. Hence, this region was defined as study area to undertake further analytical work. The collected raw materials from these two regions were used as geological comparative samples for petrographic, mineralogical and geochemical analyses. Additionally, the geological composition of secondary deposits close to the shore was studied and compared with the archaeological material, which apparently derived from one distinct beach source.

### Leirvigen 1 ballast flint (LBF)

**Fig 1:**
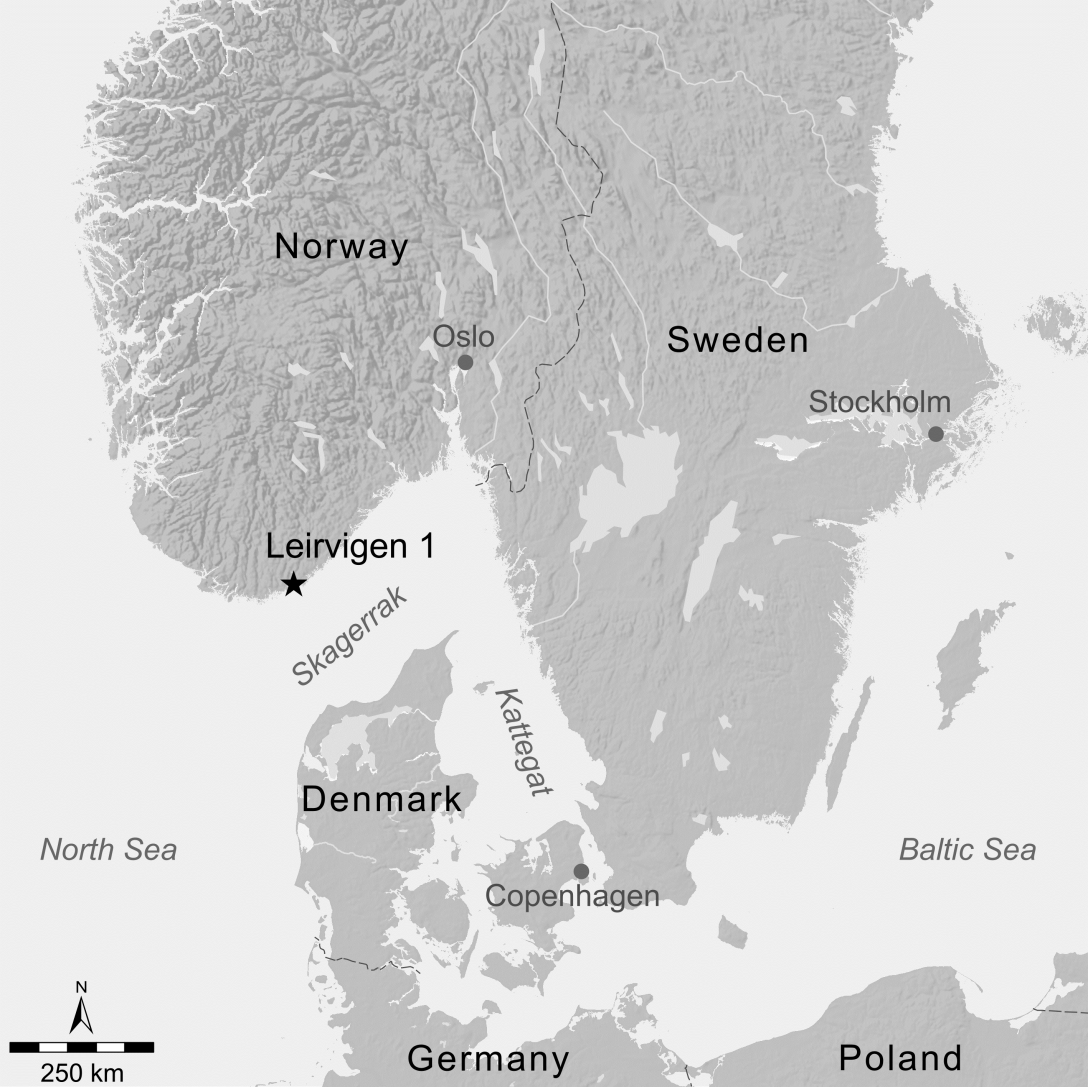
Geographical map with the location of the Leirvigen 1 shipwreck. Coolen J, Brandl M.

The archaeological site is located close to the Southern coast of Norway and recorded as Leirvigen 1 (Fig 1). Radiocarbon investigations of the ship`s wood produced C^14^ dates ranging from cal AD 1455–1620.

The Leirvigen wreck was unfortunately broken due to dredging, but the conserved parts show that it was a flat-bottomed ship with carvel construction at the bottom and clinker built sides. The flint was found within the preserved ship’s parts and can be interpreted as the vessel’s ballast or cargo [32]. A representative sample of the well-rounded ballast boulders, indicative for an origin from a secondary beach deposit, is now housed at the Norwegian Maritime Museum in Oslo. Most importantly, this assemblage constitutes a single deposition event and the raw material of the ballast stones is highly uniform, i.e. they were gathered from a single source and are therefore suitable for provenance studies. These advantageous preconditions encouraged this large-scale provenance study of Scandinavian flint varieties in order to locate the source of the Leirvigen 1 ballast flint (LBF).

### Geological material—Scandinavian flint

The term “flint” is a critical one due to its inconsistent use by both, geologists and archaeologists, in relation to the term “chert”. While both words are sometimes used interchangeably, they are in other cases understood as two separate materials. Additionally, in some regions flint is defined as a variety of chert, or vice versa [33–35]. Traditionally, in American and British literature flint is commonly understood as a nodular variety of chert of Upper Cretaceous age [36–38].

However, outside this debate in North America and Great Britain, an entirely different distinction is made chiefly based on quality. Materials with excellent knapping properties, e.g. from the Lessini Mountains or the Gargano area in Italy, from the vicinity of Kraków, Krzemionki Opatowskie or the Holy Cross Mountains in Poland, the extensive sources of the Moesian platform in Bulgaria (the famous “Balkan flint”), and many more are frequently classified as “flint”. In contrast, silicites of “poorer” quality in these regions are consequently identified as “chert” [39].

There is to date no all-encompassing solution for this terminological dilemma. Thus, we decided to apply the concept proposed by Přichystal [39] and detailed by Brandl [40] that flint in the true sense is a variety of chert and has comparable fossil inclusions and formation environments based on its

1. exclusive occurrence in Europe north of the Alps and most commonly in Scandinavia, with flint-bearing formations extending from England to western Russia;
2. Upper Cretaceous (including Danian) age; and
3. distribution in distinct host rock facies (i.e. Cretaceous chalk and Danian limestone).

In agreeance with Högberg and Olausson [1], the material dealt with in the current contribution will be referred to as “Scandinavian flint” to distinguish it from other flint types, such as “southern” flint from France, Belgium and the Netherlands [41].

## Geology of the study area

**Fig 2:**
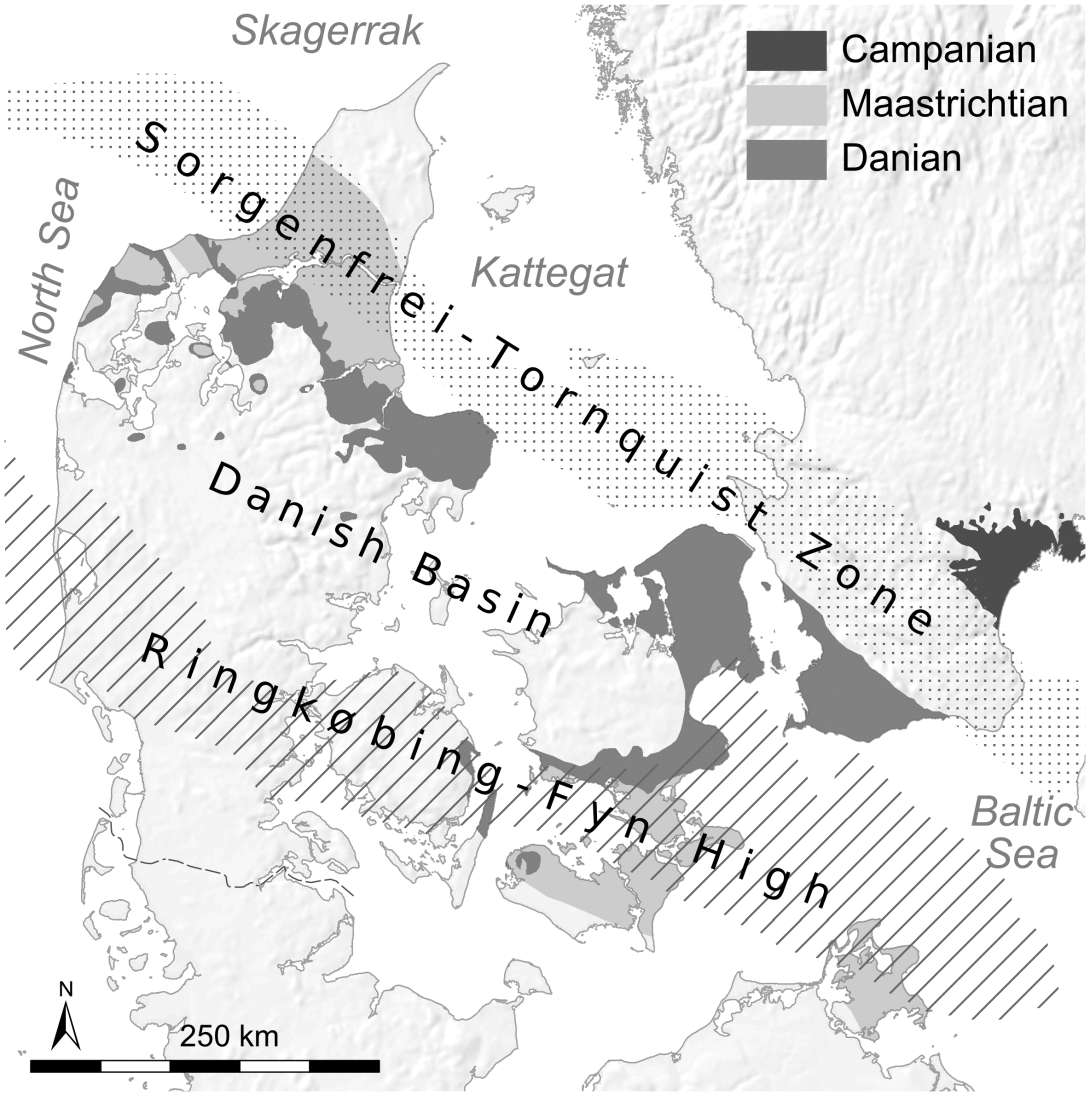
Simplified geological map of the study area. Coolen J, Brandl M.

The study region is located within the Danish parts of the north-west European “Chalk Sea”. This area is dominated by the Danish Basin, bordered by the Sorgenfrei-Tornquist Zone (STZ) to the northeast and the Ringkøbing–Fyn High (RFH) to the southeast (Fig 2). Upper Cretaceous (Maastrichtian) chalk was deposited in varying depths in the NW European Epeiric Sea, but always well below the photic zone and storm wave base. Chalk deposition reached a thickness of over 2000 meters in the Danish Basin and extended in a belt along the STZ. Over structural highs such as the RFH and the STZ, the chalk thickness is significantly reduced due to erosional processes [42]. Within these extremely pure chalk deposits formed distinct layers of flint nodules.

During the Paleocene, Bryozoan wackestone and packstone mound complexes containing flint layers and nodules were deposited below the photic zone on the deep water shelf of the Danish-Polish Trough in a depth between 100 and 300 meters [43, 44].

### Flint formation

Directly linked to the NW European Late Cretaceous chalk and early Paleocene (Danian) limestone formations are the occurrence of flint layers and nodules. Flint did not form directly on the sea-floor but at a distance beneath the sediment surface, indicating original sea-bottom contours and hence successive sequences of sea-bottom topographies [45].

Recently, a model for the formation of flint in the North Sea chalk was proposed [46, 47]. Following this model, nano-quartz particles (i.e. α-quartz spheres with diameters of ~500 Å) were deposited on the sea floor together with bioclastic material. Due to the colloidal nature of the nano-quartz crystals, a strong flocculation tendency was observed, which is an essential precondition for the formation of flint layers. As a consequence of acidification of the sea water related to volcanic activities during the Late Cretaceous and Early Paleogene periods, practically all calcitic bioclasts were dissolved in some areas, resulting in high concentrations of nano-quartz particles triggering flint formation.

The most important question for flint analyses involving geochemistry concerns the origin of silica (Si). Lindgreen et al. [46] propose the origin of Si from dissolved microorganisms such as radiolarians and diatoms in deep sea conditions, and from sponges in more shallow water shelf environments. Volcanism in the form of hydrothermal activities in the North Sea region has to be considered as an additional important silica source [48, 49].

## Hypotheses and scientific questions

The working hypothesis for the present sourcing study suggests that depositional basins are characteristic and discernable from each other in their composition of the microfauna as well as geochemical signatures. Based on the geographic position of individual outcrops in the study region we tested possibilities to reconstruct such paleobasins in which flint was formed to define distinct flint provinces. Within those flint provinces we attempted a more refined source separation with increasing geographical resolution based on specific geological settings, generating a “fingerprint” of each individual source. Subsequently, the archaeological material was investigated in order to trace it back to its original source.

The Scandinavian flint provenance study is structured in four consecutive steps:

1. The initial step focused on a differentiation between sources of Late Cretaceous (Maastrichtian) and Early Paleocene (Danian) age throughout the study region.
2. In a second step, we attempted to discern distinct source clusters within those larger sedimentary regimes (i.e. the Maastrichtian and the Danian flint provinces) with the assumption that such source clusters would be representative of submarine depositional basins in which flint was formed.
3. Eventually the potential to discriminate between particular sources within one of the defined clusters (i.e. depositional basins) was tested.
4. Ultimately, a representative number of archaeological material (i.e. samples from the Leirvigen 1 ballast flint) was contrasted against analytical results from the primary geological samples and the composition of beach deposits in order to assign the LBF to one of the identified source areas and to establish the provenance of the archaeological material.

## Materials and methods

### Geological samples

Additional to a characterization of primary flint deposits, the archaeological case study attempted to source samples from secondary deposits. Such undertakings are generally considered problematic [50], however, previous studies involving geochemistry have demonstrated the potential of such efforts [51]. Our sampling strategy was based on the argument that materials from secondary sources inherently bear the closest similarities in microfossil content and trace element distribution to materials from their primary geological host environments [4]. Thus, once a reliable fingerprint has been established for primary deposits, it should be possible to assign samples from secondary deposits to a primary source area. Following this strategy, we investigated 13 primary and 37 secondary sources in the study region. The primary geological localities, five of Maastrichtian- and eight of Danian age, were chosen from previous studies that established a comprehensive groundwork for the current undertaking [1–4].

**Fig 3:**
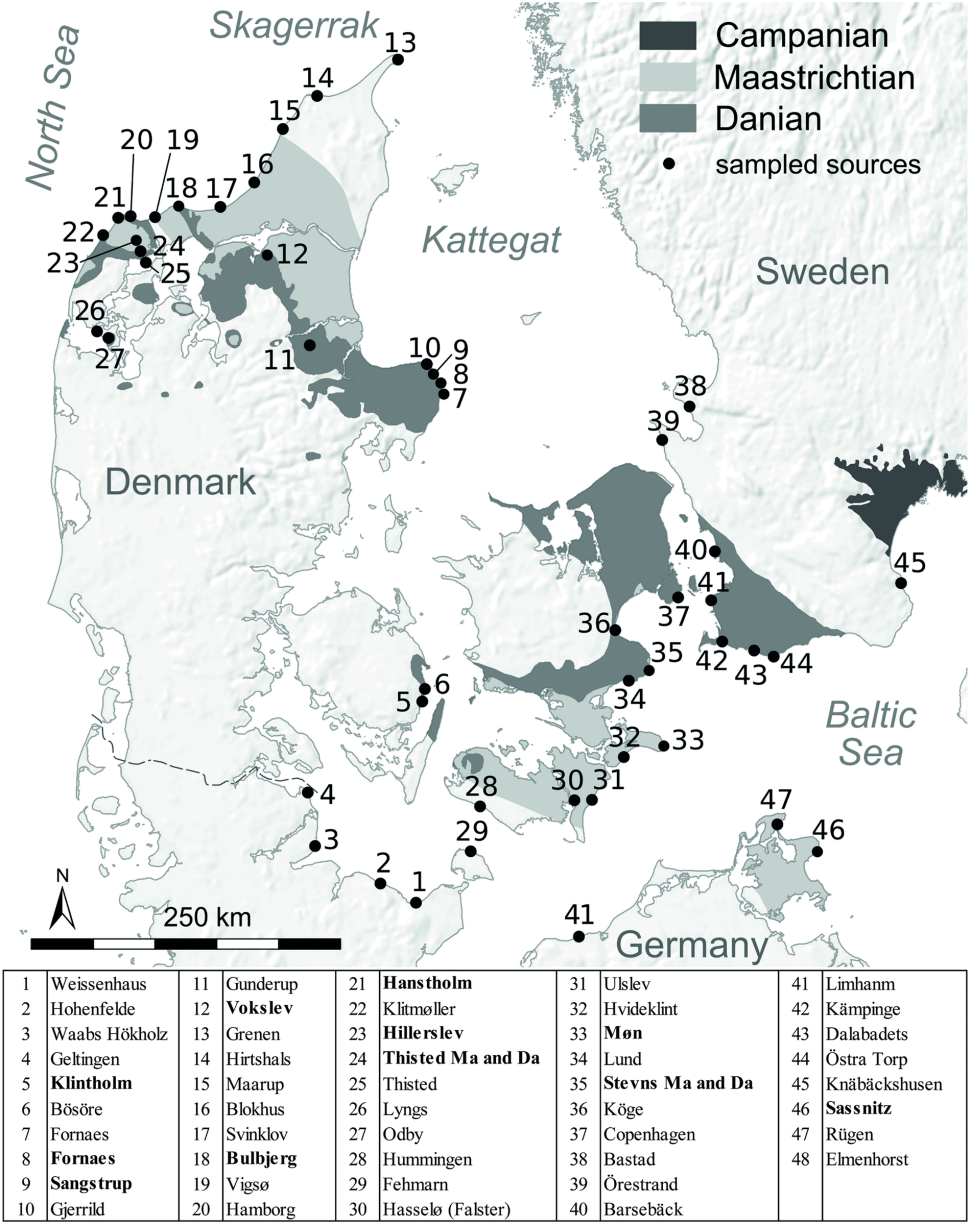
Primary and secondary deposits used for this study. Coolen J, Brandl M. Thisted and Stevns were sampled twice (STMa/STDa and THMa/THDa).

Maastrichtian flint samples were collected from Hillerslev (HI), Thisted (THMa), Stevns (STMa), Møn (MO) in Denmark and Sassnitz (SN) from Rügen in Germany. Flint deposits of Danian age were sampled at Bulbjerg (BU), Hanstholm (HH), Vokslev (VO), Thisted (THDa), Sangstrup (SA), Fornæs (FO), Stevns (STDa) and Klintholm (KH), all located in Denmark (Fig 3).

The chalk from which all Danish Maastrichtian flint samples were collected corresponds to the uppermost section of the North Sea Chalk Group, the Tor formation [52]. Flint samples from Hillerslev and Thisted in Jutland and samples from Møn derive from the upper part of this geological unit. At Stevns, a flint seam from the upper Sigerslev member was sampled. Litho-stratigraphically, flint samples from Sassnitz in Germany belong to the late Lower Maastrichtian Rügen member of the Hemmoor formation. All sampled limestone outcrops of Danian age correspond to the Stevns Klint Formation [43, 53].

From each source, 20 individual flint nodules from one distinct layer clearly assigned to a geological formation were chosen for characterization. Given the wide range of visual variation of Scandinavian flint types [2], the flint variety dominating each sampled locality was chosen for analyses in order to produce significant and reproducible results. Altogether, 260 individual samples from secure lithostratigraphic context were analyzed.

Since the LBF clearly derived from a secondary context, 37 secondary deposits were selected according to a “coherent sampling scheme” [33]. For this task, beach deposits within the entire study area including South Sweden were sampled in a distance of approximately 50 km to investigate the varying composition of such deposits and to contrast the results against those from the primary outcrops.

### Archaeological samples

**Fig 4:**
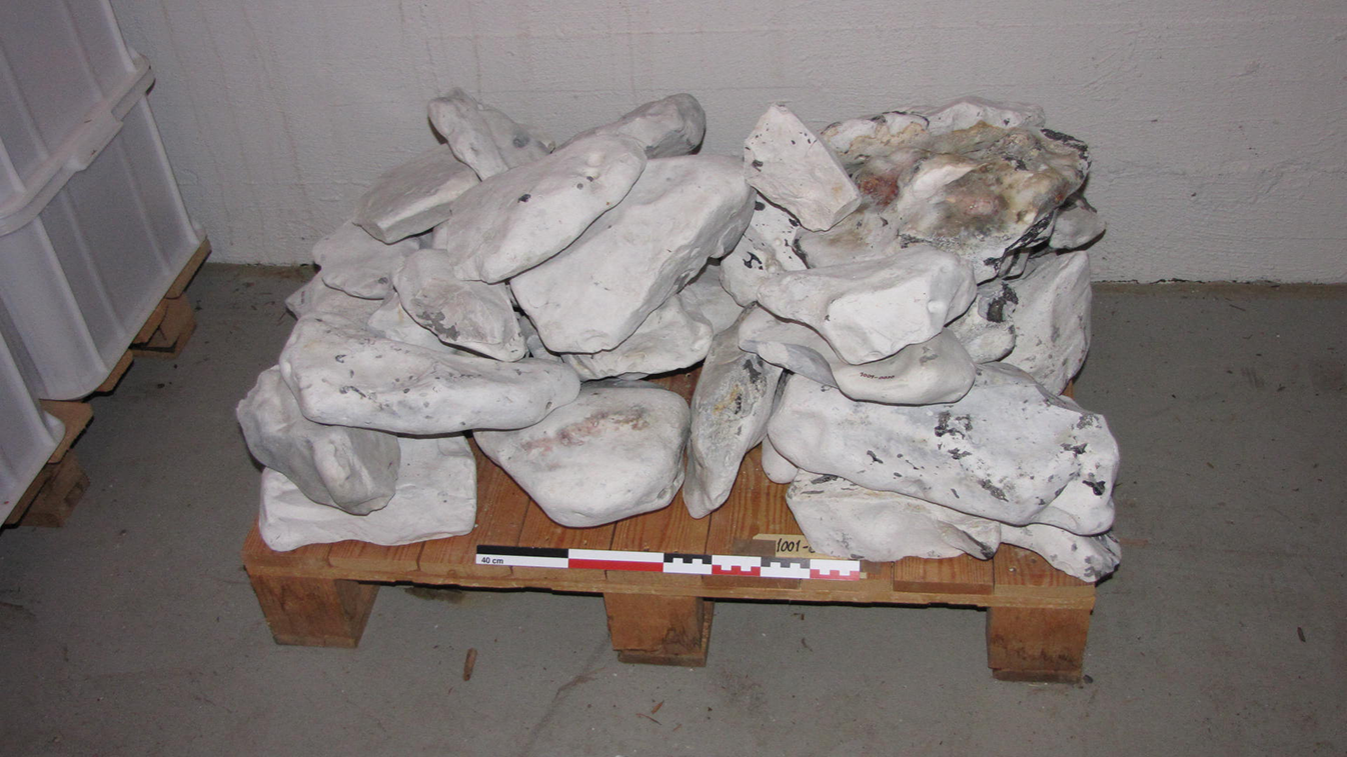
Sample of Leirvigen 1 ballast at the Norwegian Maritime Museum in Oslo.

The lithic ballast material recovered from the Leirvigen 1 shipwreck is stored at the Norwegian Maritime Museum in Oslo (reference no. 10010030 for all samples) and consists of 30 naturally rounded flint-bearing limestone boulders ranging between 15 and ca. 40 cm in diameter (Fig 4). From this assemblage, 20 individual specimens were chosen for provenance studies. For geochemistry, flint inclusions in the limestone boulders which were sufficiently large and homogeneous and thus considered representative were mechanically extracted. For microfacies analysis, larger samples of limestone containing flint were knapped off 5 of the investigated boulders in order to test if the material corresponds to only one or several lithological contexts.

### Analytical strategy

Samples from beach deposits were studied according to macroscopic flint variety distribution, grain size (i.e. sand—gravel—cobble—boulder), sorting and degree of erosion. All samples from primary contexts were analyzed according to the Multi Layered Chert Sourcing Approach (MLA) combining visual comparative studies, stereo-microscopic analyses of microfacies and geochemical trace element analyses using LA-ICP-MS (Laser Ablation Inductively Coupled Plasma Mass Spectrometry) for source area identification and separation [6].

#### Visual (macroscopic)

Macroscopic inclusion patterns and the range of color according to Munsell were investigated. During this analytical stage, distinct raw material groups can be separated and microscopically and geochemically tested for their consistency [33, 54, 55].

#### Microscopy

Reflected light microscopy allows for the detection of trace fossils and non-fossil inclusions in silicites. Micropaleontological and microfacies analyses are useful tools to determine the age and genetic environment of silicites. Sedimentary facies can be differentiated based on texture and components. Texture represents the relation between the groundmass (matrix) and the components of a rock. Criteria for facies identification are matrix type and abundance and distribution of the components, typically fossil and non-fossil inclusions [44]. Both classification systems for carbonate rocks proposed by Dunham [56] and Folk [57, 58] were used for characterization. Microscopic studies were performed at 20 individual macrosamples for each source. For primary feature analyses a biological stereomicroscope with up to 70 x magnification was used. In order to make our results applicable to archaeological finds, which in most cases require non-invasive analyses, all micropictures for this study were produced on unpolished rock surfaces qualitatively illustrating source characteristic inclusion patterns at 40 times magnification and under water immersion. For petrographic investigations of specific mineral phases in selected flint samples, polished surfaces were analyzed by SEM-EDX-WDX (Jeol JSM 6310, Institute of Earth Sciences, Graz University) and with a Petrographic Zeiss Axio binocular with Canon EOS 750D camera application.

#### Geochemistry

Instrumental sourcing techniques are increasingly gaining importance in archaeological research, however geochemistry is still only occasionally employed for lithic provenance studies [59–62]. In the last decades, various geochemical methods have been employed for sourcing chert and flint with varying success [4, 63–65]. Considering the comparatively heterogeneous nature of siliceous rocks and the typically very low trace element concentrations in such materials [33, 66, 67], LA-ICP-MS is increasingly used for their chemical characterization [59, 60, 62, 68–73]. LA-ICP-MS requires only minimal sample preparation and allows for the rapid simultaneous detection of up to 50 elements at highest sensitivity [74–76]. Successfully working with this technique requires to comprehend the complex geochemical processes responsible for the distribution of trace elements in siliceous rocks, which can be outlined as follows:

1. Silicon (Si^4+^) can be substituted (i.e. replaced) in the crystal lattice of chert and flint by other cations with a similar ion radius and charge. These typically immobile elements include aluminum (Al^3+^), titanium (Ti^4+^), germanium (Ge^4+^), iron (Fe^3+^) and phosphorus (P^5+^). A charge difference of 1 is possible, however additional cations or crystal defects are necessary to establish neutrality in the system [77]. Although trace element substitution in the crystal lattice of quartz is not very common, such generally immobile elements can be used to achieve a differentiation between chert and flint sources.
2. Chemical elements can be incorporated into the lattice interstitials and pore spaces of silicites. In the case of aluminum (Al^3+^) and iron (Fe^3+^), additional cations such as lithium (Li^+^), sodium (Na^+^) and hydrogen (H^+^) can occupy interstitial positions in the crystal lattice. Deposition of trace elements can occur cogenetically during sedimentation or through secondary processes in the course of diagenesis [78]. Cations situated in the lattice interstitials and in pore spaces during the formation of SiO_2_ modifications include strontium (Sr), vanadium (V), rubidium (Rb), barium (Ba), boron (B) and lithium (Li) and are in many cases suitable for a source discrimination. Conversely, alterations of the rock surface after the material was formed can significantly change the chemical composition by depletion or enrichment of elements [4, 79]. Most commonly, this involves coloring cations such as iron (Fe), manganese (Mn), chromium (Cr) and nickel (Ni), resulting in darker or polychrome areas on rock surfaces. These effects are also known as “patination” and have to be avoided for geochemical analysis.3. Synsedimentary (i.e. during the process of sedimentation) inclusion of foreign minerals commonly involves feldspar, carbonates, clay minerals and heavy-minerals (e.g. rutile, hematite, magnetite, etc.). Trace elements calcium (Ca), aluminum (Al), potassium (K), iron (Fe), manganese (Mn), nickel (Ni), chromium (Cr), barium (Ba), magnesium (Mg), strontium (Sr), vanadium (V) and rubidium (Rb) can be enriched in such inclusion minerals [80]. Specifically calcium in combination with aluminum, magnesium and strontium reveals the genetic environment of chert and flint and consequently the origin of trace elements, e.g. from carbonates, plagioclase or clay minerals. Elements related to inclusion minerals are suitable for provenance studies if they are source specific, i.e. if their distribution is unique at each source location. In the case of coloring cations such as Fe and Mn it has to be ensured that they are not secondarily enriched in the course of postgenetic alteration processes.

For the present study, Laser Ablation Inductively Coupled Plasma Mass Spectrometry (LA-ICP-MS) analyses were performed with an Agilent 7500ce quadrupole ICP-MS unit located at the Central Lab for Water, Minerals and Rocks, NAWI Graz Geocenter (University of Graz and Graz University of Technology, Austria), with sample introduction through an ESI NWR-193 laser ablation system. Effects of naturally occurring heterogeneity within sedimentary rocks were controlled and minimized by analyzing each sample at three discrete spots, resulting in 780 multi-elemental measurements for geological samples, and 66 for the archaeological material. Geochemical data of 36 elements, which were found to be useful in earlier provenance studies [70, 73], were effectively determined (S1 Table). Small chips of (c. 2 mm) of both, geological and archaeological samples, were imbedded into resin mounts and polished in order to avoid analyzing chemically altered rock surfaces (“patination”). The spot size of the 193 nm wavelength laser was 75 μm, operated at 10 Hz pulse frequency corresponding to an energy of ~8 mJ cm^-1^. Ablated material was transported via a helium gas stream (0.7 1 min^-1^) into the argon plasma torch of the mass spectrometer and passed into the ICP-MS unit. Standard reference glass NIST SRM 612 was routinely analyzed for standardization and drift correction (concentrations from [81]). NIST SRM 614 was analyzed as unknown and reproduced within 10% relative error. Silicon (Si) was used as internal standard. For data reduction in GLITTER, a SiO_2_ value of 99 wt% was established for Scandinavian flint. Detection limit of LA-ICP-MS is typically 0.01–0.1 ppm for most elements, however values below 1 ppm need to be treated with caution since microinclusions, which are commonly occurring in flint and chert samples, might influence the concentration of the element of interest significantly at this concentration level.

#### Statistical evaluation

Bivariate scatter plots are used to reveal individual elements which can be considered source specific, and hence responsible for a differentiation of geological sources using trace element concentrations. Such elements allow the reconstruction of geological formation processes and indicate why a differentiation can or cannot be achieved. However, standard statistical methods cannot be used for further data analyses, since they produce biased results when applied to raw geochemical data (i.e. absolute measured values). Hence, we evaluate multivariate geochemical datasets by Compositional Data Analysis (CODA).

For the present study we used stepwise variable selection, with X representing the complete original data matrix:

1. Apply LDA on ilr(X) and compute the CV-misclassification rate (mcCV).
2. Apply LDA on ilr(X[,-j]) (remove j-th variable), for j = 1, …,#columns of X, and compute mcCV for each j. Remove that variable for which the largest reduction of mcCV is achieved, but only if this results in a smaller mcCV as for the previous step.
3. Proceed as in 2) with removing one variable at a time, as long as mcCV can be reduced.

## Analytical results

### Geological samples

#### Macroscopic (visual) and microscopic analyses

On a macroscopic scale, Maastrichtian samples typically appear darker than Danian specimens and display more or less frequent grey intraclasts in a homogeneous rock matrix. All Danian samples are characterized by high porosity linked to minute cavities derived from poorly silicified macro- and microfossil inclusions (mainly bryozoan skeletons and “calcispheres”), resulting in a heterogeneous rock texture. Based on the work from Högberg and Olausson [1], Högberg et al. [2] and the results of the present study, it is possible to propose a systematic classification of visually distinct flint varieties considering geological age and visual characteristics (Table 1). However, these types are not source specific, and macroscopic source assignment is therefore not possible.

**Table 1:** Classification of flint types based on geological age and visual characteristics.

geological age	visual characteristics	short code	eponym Subtype	primary locations
Maastrichtian	Black Flint	MBF	Stevns/Hov	Hillerslev, Thisted, Ellidshøj, Stevns, Hvideklint, Sassnitz, Bjerre, Hov, Sallerup
Maastrichtian	Gray Flint	MGF	Møn	Mön, Sassnitz
Maastrichtian	Speckled Flint	MSF	Gøl/Møn	Hillerslev, Møn, Sassnitz
Maastrichtian	Banded Flint	MBdF	Falster	Falster
Danian	Bryozoan Brown Flint	DBB	Funen	Fornæs; Sangstrup, Bulbjerg, Hamborg, Thisted, Vokslev, Stevns, Faxe, Klintholm, Limhamn
Danian	Bryozoan Gray Flint	DBG	Funen	Klintholm
Danian	Bryozoan Black Flint	DBBl	Funen	Klintholm
Danian	Gray Banded Flint	DGB	-	Hanstholm, Vokslev, Ellidshøj
Danian	Gray Matte Flint	DGM	Östra Torp	Östra Torp
Variegated flint varieties (not detected at primary sources)		SVF	-	glacial tills around the Baltic Sea

Principal microscopic parameters for source differentiation are type and abundance of fossil and non-fossil inclusions (S2 Table). For direct comparison, only microscopic features considered useful for source identification were recorded, and inclusion abundance in the rock matrix was averaged [82]. There exist recognizable differences in trace fossils between individual deposits of the same geological age, however they do not appear to be consistent enough to allow secure assignments to a specific outcrop [83]. A general differentiation is possible between Maastrichtian and Danian samples, mainly based on the significantly higher abundance of Bryozoan inclusions in Danian samples. Conversely, Maastrichtian flint is richer in shell-, brachiopod- and echinoderm remains due to specific diagenetic conditions directly affecting the preservation of microfossil inclusions, especially shell structures. Non-fossil inclusions were also detected in the course of SEM analyses, which revealed rutile, Fe-oxides, carbonates and Sulphur. Petrographic investigations under polarized light produced evidence for frequent Fe-sulfide and rarer Fe-oxide inclusions in both, Maastrichtian and Danian samples, however they are insignificantly interspersed into matrices of all investigated flint samples, and hence not suitable for source division.

#### Microfacies

All investigated Maastrichtian flint samples can be classified as fossiliferous micrite according to Folk [58], which agrees well with a slightly bioturbated pelagic depositional environment. The majority of Danian samples correspond to packed biomicrite and those from Klintholm to sparse-packed biomicrite, both in agreeance with benthic bryozoan limestone facies (S1 Fig).

#### Geochemistry

Trace elemental distribution in sea sediments chiefly depends on biogenic composition, depositional environment and geochemical milieu at the sediment-water interface. For the present study, trace elements strontium (Sr), aluminum (Al), magnesium (Mg), manganese (Mn), germanium (Ge) and rubidium (Rb) were found to be geochemically significant for bivariate analyses revealing source specific depositional and post-depositional mechanisms. For statistical analyses applying CODA, the complete data set was used with stepwise data reduction as indicated in materials and methods.

#### 1. Maastrichtian versus Danian samples: Bivariate analyses

**Fig 5:**
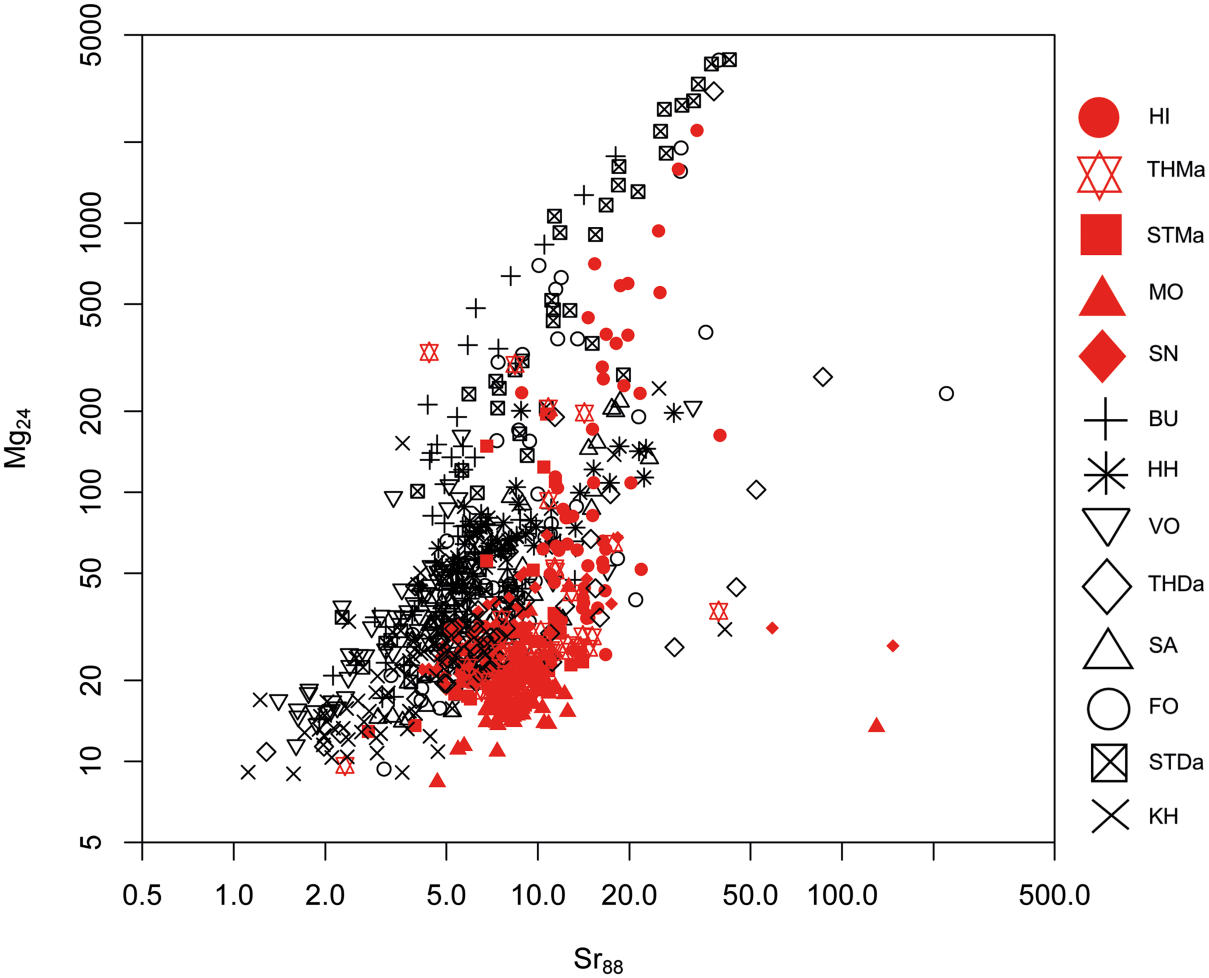
Strontium (Sr) versus magnesium (Mg) concentration plot of all geological samples.

**Fig 6:**
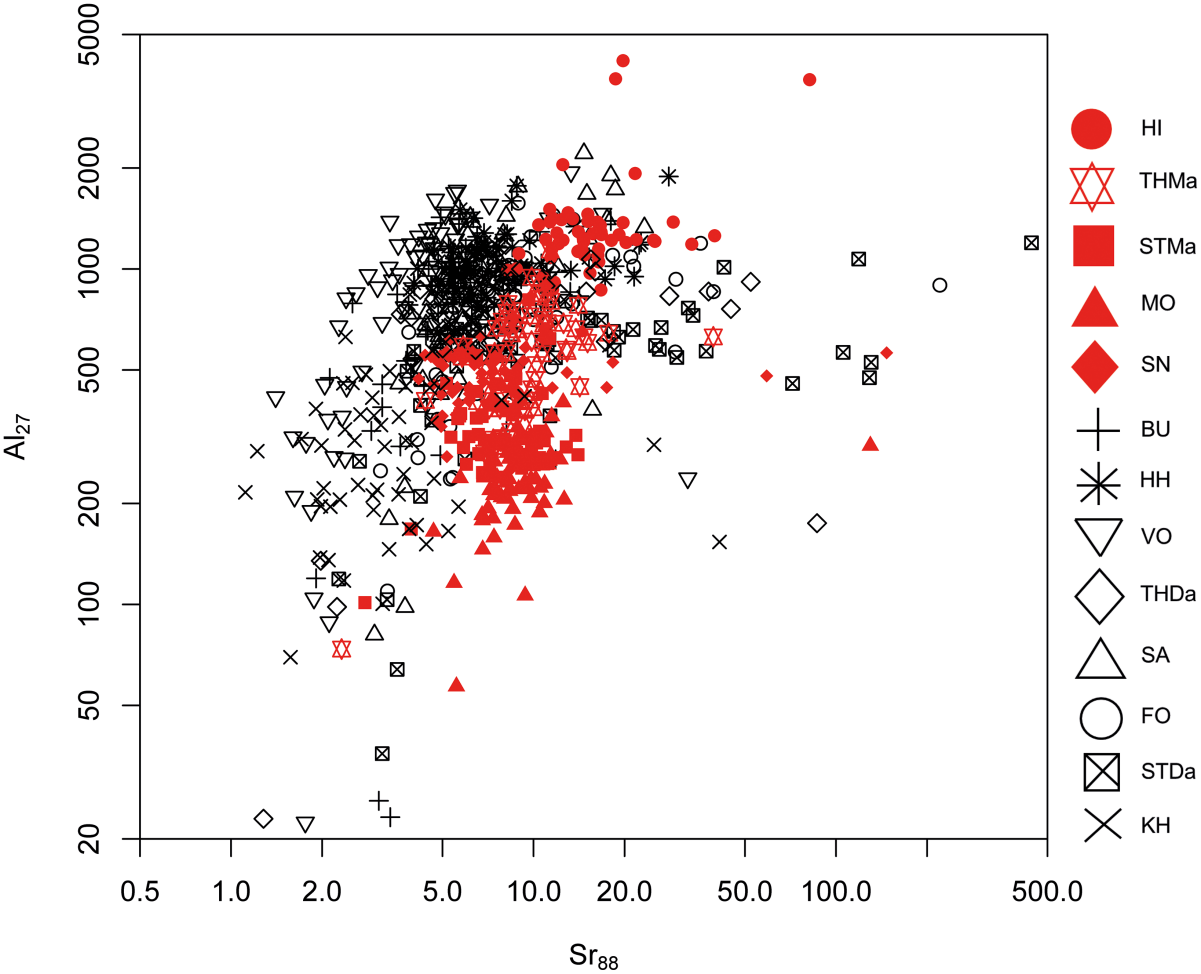
Strontium (Sr) versus aluminum (Al) concentration plot of all geological samples.

**Fig 7:**
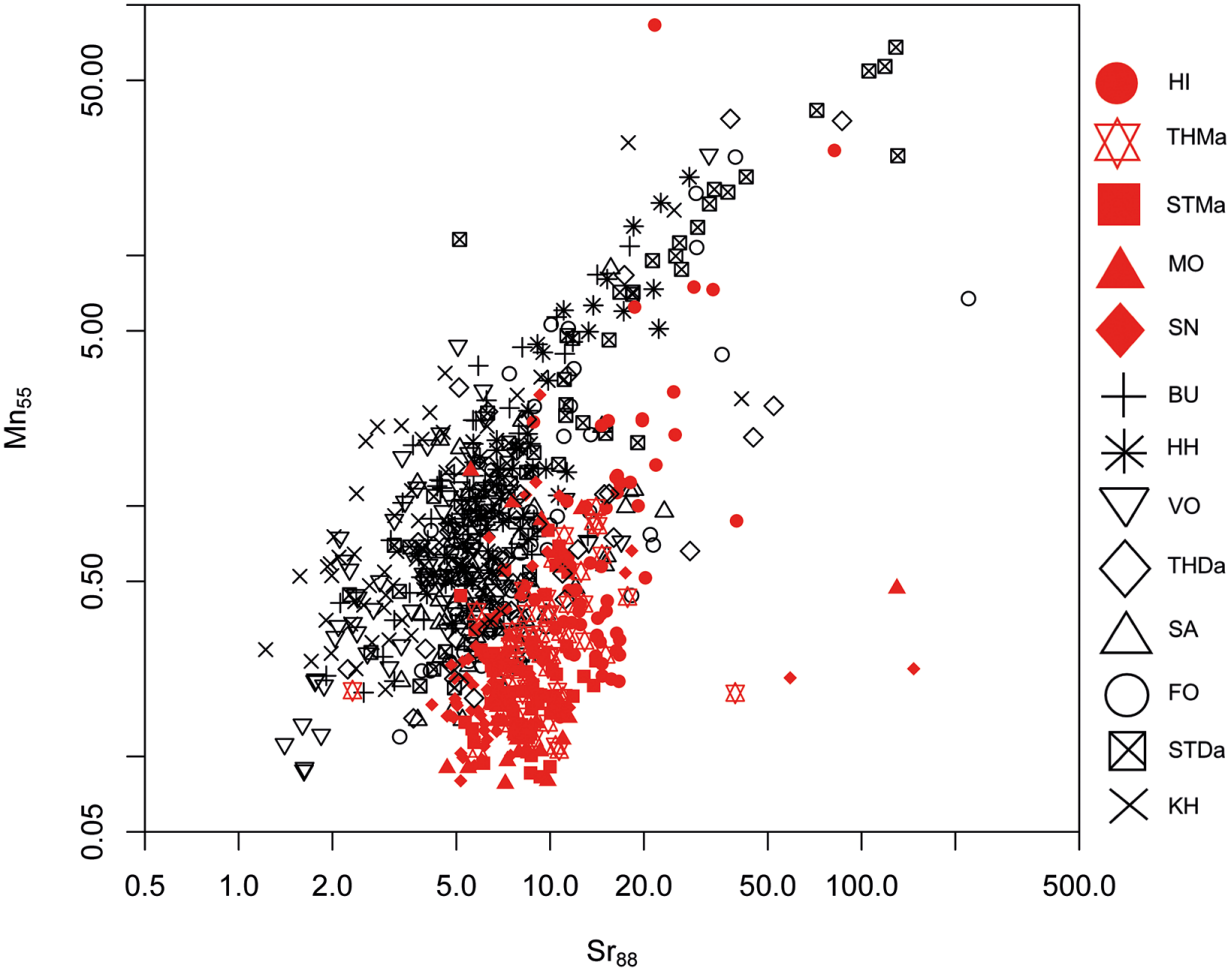
Strontium (Sr) versus manganese (Mn) concentration plot of all geological samples.

**Fig 8:**
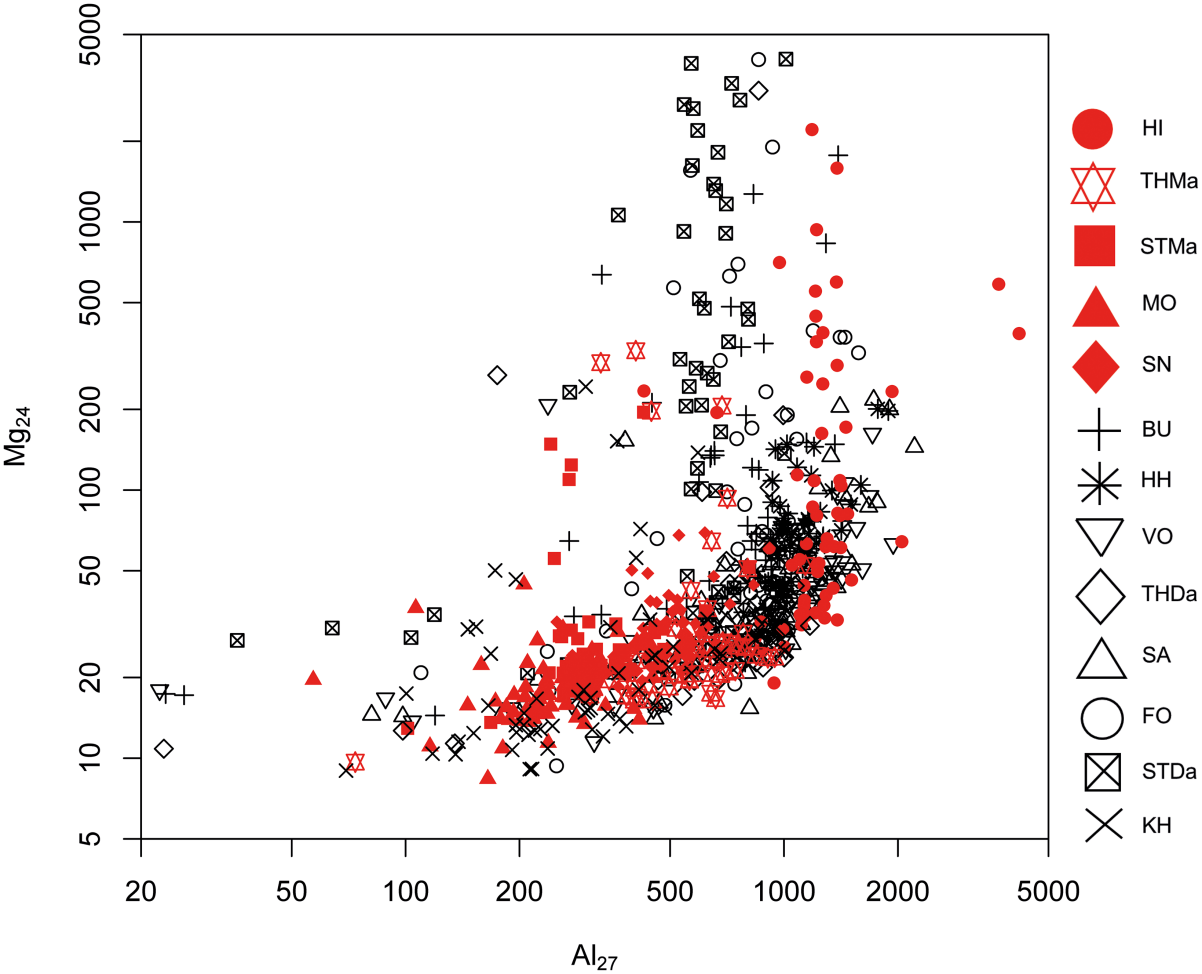
Aluminum (Al) versus magnesium (Mg) concentration plot of all geological samples.

A differentiation of the two units can be achieved to a certain degree by Sr versus Mg, Al and Mn concentrations (Figs 5, 6 and 7). However, significant overlapping effects occur. By tendency, Mg, Al and Mn are slightly enriched in Danian samples (Figs 7 and 8), whereas Maastrichtian flint generally displays higher Sr values. The variation of trace element concentrations is related to changing conditions in depositional facies, including depletion processes. In general, Maastrichtian chalk contains higher amounts of Sr and lower concentrations of Mg and Mn compared to Danian limestone, which is in agreeance with findings of Jørgensen [84] and Kunzendorf and Sørensen [85].

**Fig 9:**
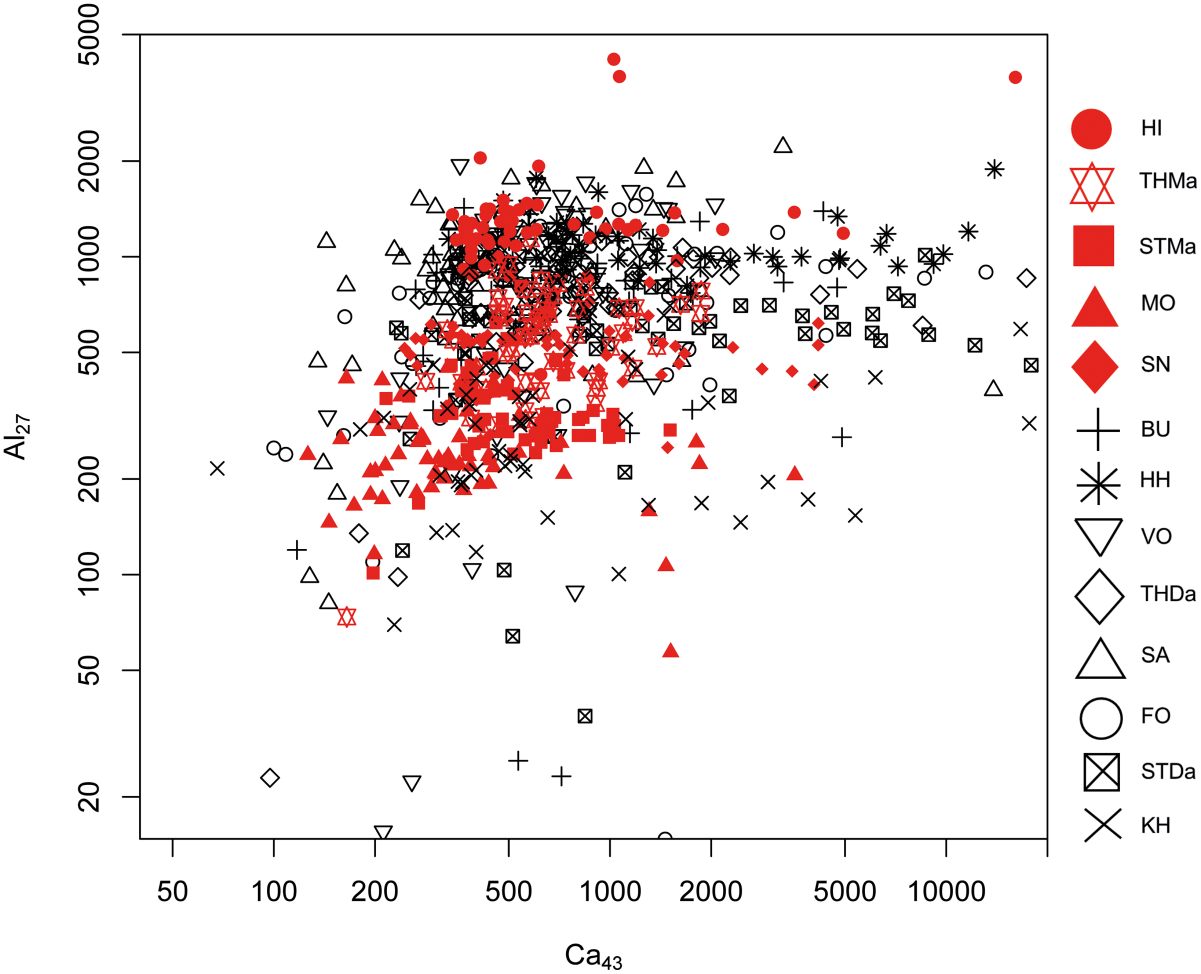
Calcium (Ca) versus aluminum (Al) concentration plot of all geological samples.

**Fig 10:**
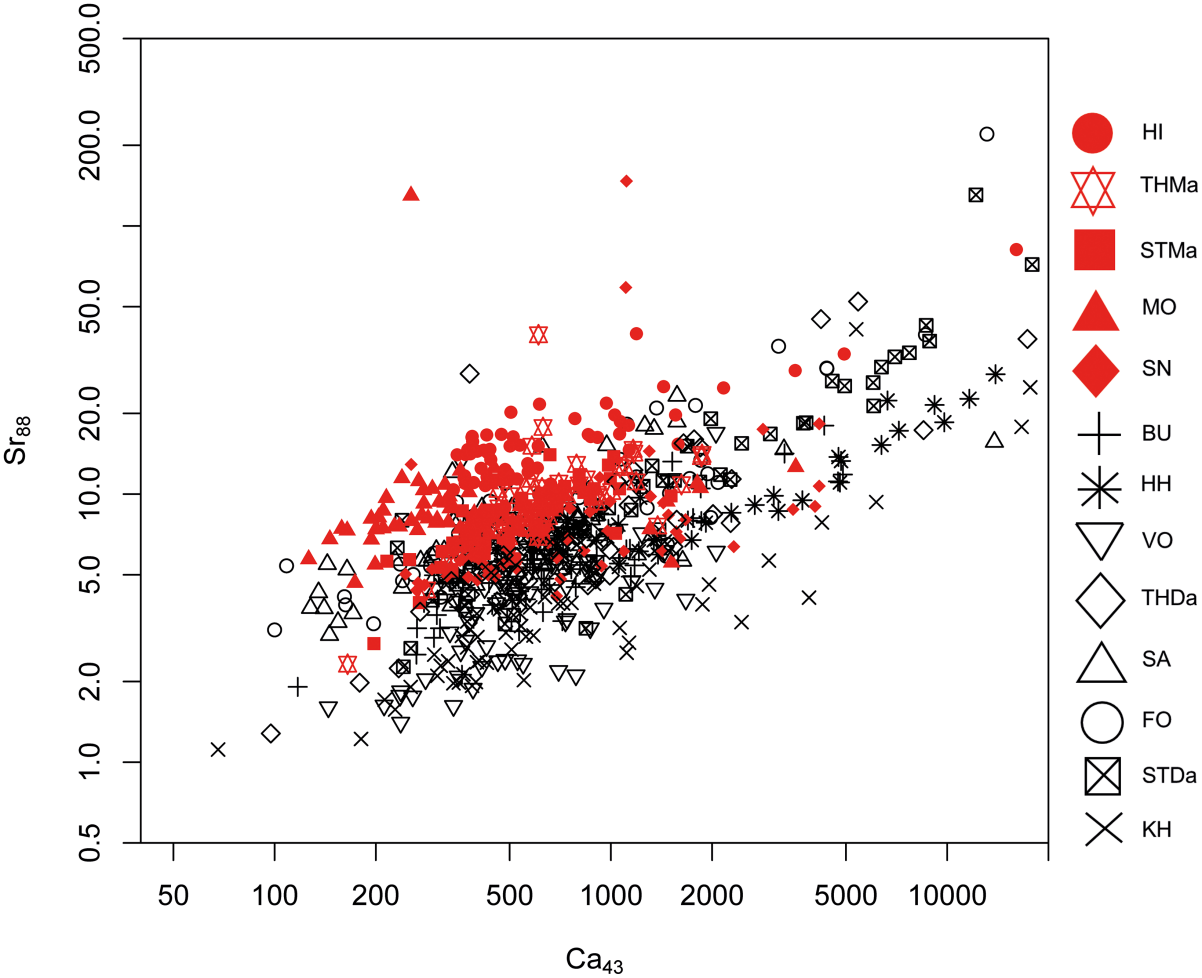
Calcium (Ca) versus strontium (Mg) concentration plot of all geological samples.

Correlations with calcium (Ca), the primary constituent of the host rock facies, are useful for reconstructing the geochemical, i.e. paleo-depositional, environment of the investigated sources, which is representative of source-genetic conditions. High Ca concentrations are present in HI (Maastricht), and HH, STDa, and some KH (Danian) samples (Figs 9 and 10). Generally, Danian flint displays a wider range of Ca contents. Calcium concentration may be related to carbonate phases in the silicite, however, in correlation with Al this indicates the presence of the calcic feldspar plagioclase, which may be the case in the investigated Maastrichtian chalk—Danian bryozoan limestone sequence (Fig 9). Calcium is correlated with Sr values in Maastrichtian and Danian samples (Fig 10). In the Ca–Sr correlation plot (Fig 10), all samples follow a 45° tendency displaying the similar geochemical behavior of both elements and indicate a higher or lower abundance of carbonates and/or plagioclase.

#### CODA results

1. List of variables which are removed, together with the achieved mcCV:

**Table:** 

**Rb85**	**Ce140**	**Pb208**	**Be9**	**Ti49**	**Ga71**	**Y89**
0.06410256	0.06025641	0.05641026	0.05641026	0.05641026	0.05641026	0.05641026
**Cr53**	**V51**	**Ba137**	**Zr90**	**La139**	**Sm147**	**Gd157**
0.05641026	0.05641026	0.05512821	0.05512821	0.05512821	0.05512821	0.05512821
**Nd146**	**Er166**	**Yb172**				
0.05512821	0.05512821	0.05512821
2. List of variables that remain for LDA:
  "Li7" "B11" "Mg24" "Al27" "Ca43" "Mn55" "Fe56" "Co59" "Ni60" "Cu65" "Zn66" "Ge74" "Sr88" "Cs133" "Pr141" "Eu153" "Dy163" "Th232" "U238".
3. LDA with remaining list of variables:
  mcCV = 0.05512821
  Group (grp) assignment:

**Table:** 

grp	1	2
1	**458**	22
2	21	**279**

**Fig 11:**
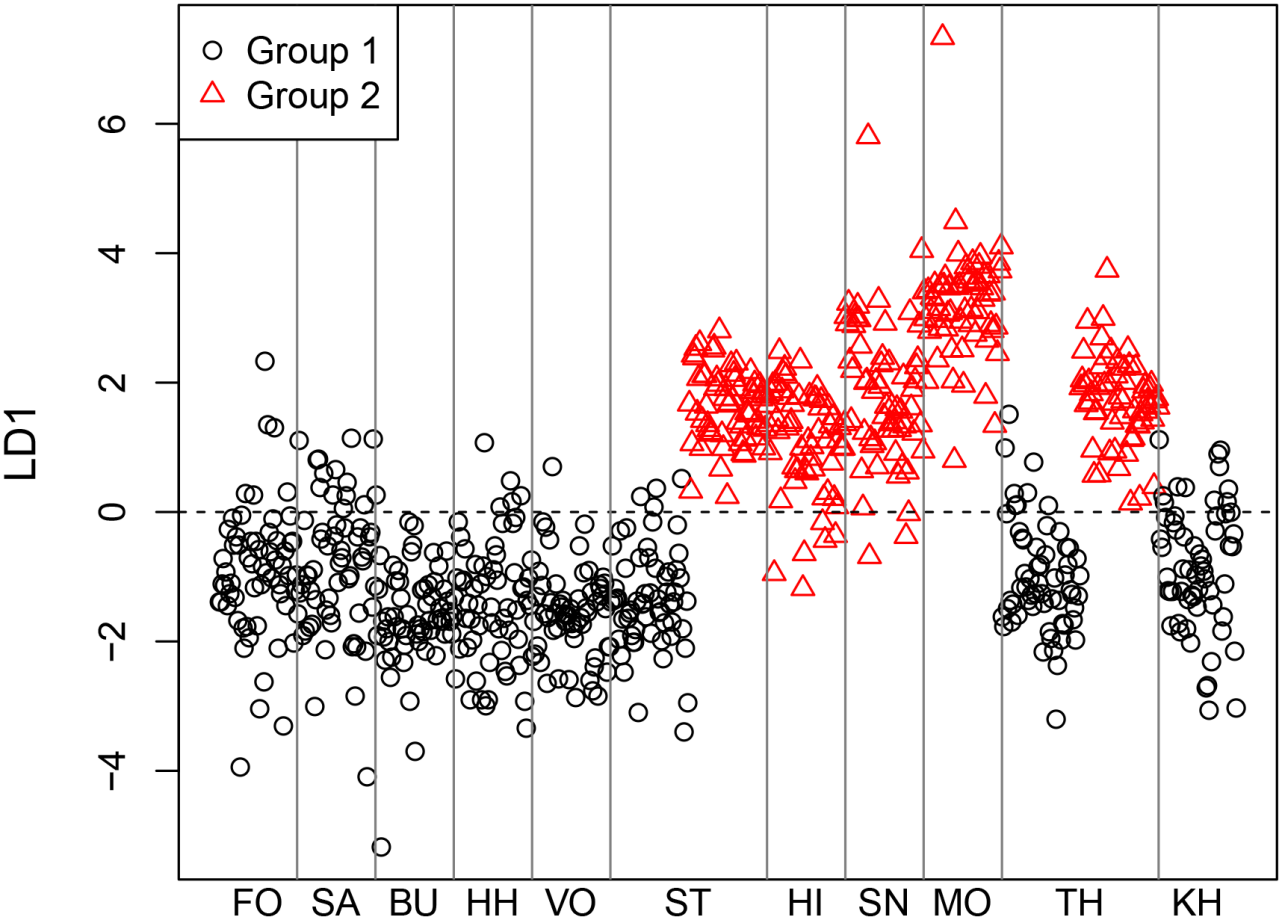
Linear discriminant plot of all geological samples.

The results of statistical data evaluation illustrate distinct group separation between Maastrichtian and Danian samples with minimal misclassification (Fig 11). Hence, an assignment of any sample to one of the two groups is possible with high reliability.

#### 2. Maastrichtian samples: Bivariate analyses

**Fig 12:**
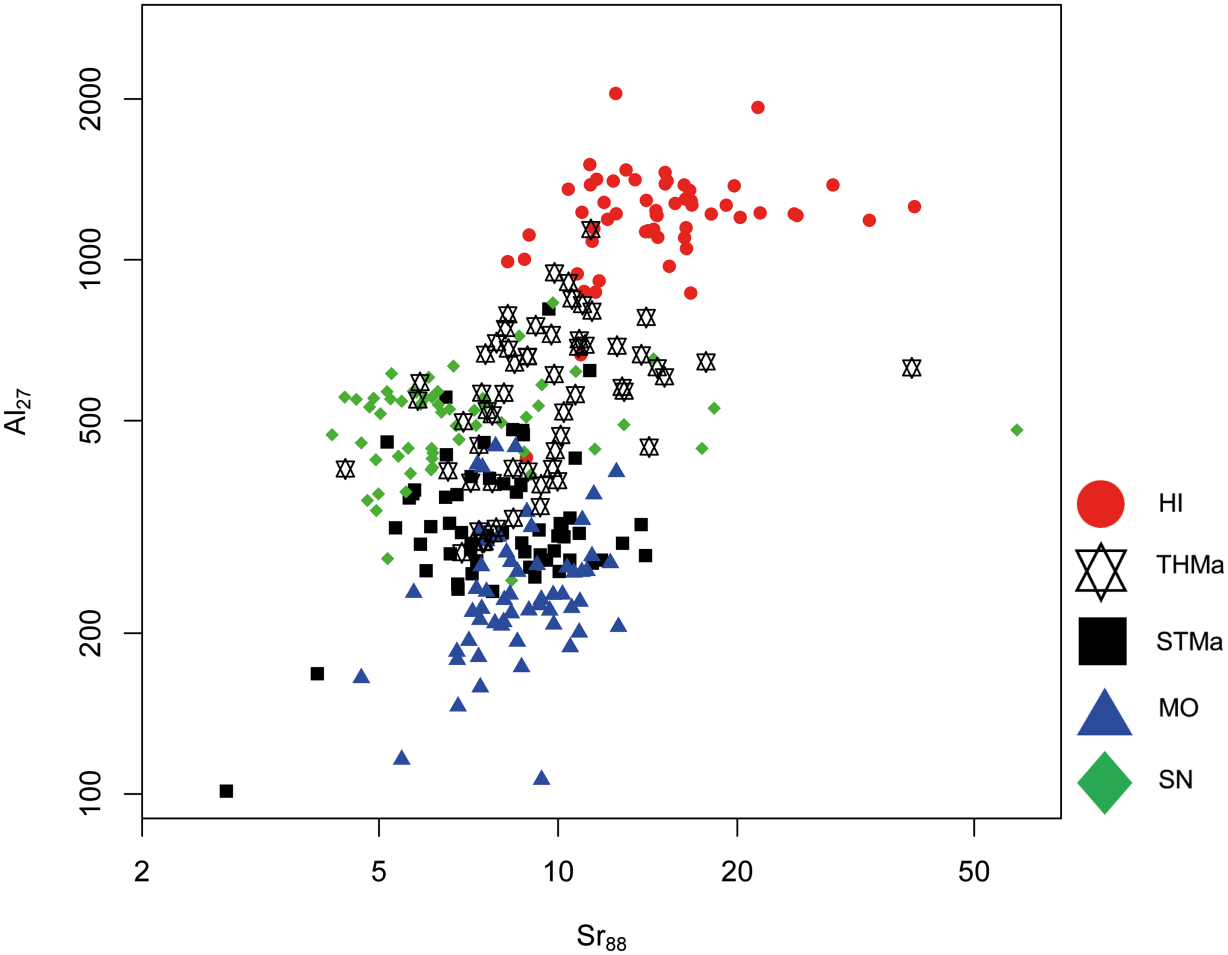
Strontium (Sr) versus aluminum (Al) concentration plot of all Maastrichtian geological samples.

**Fig 13:**
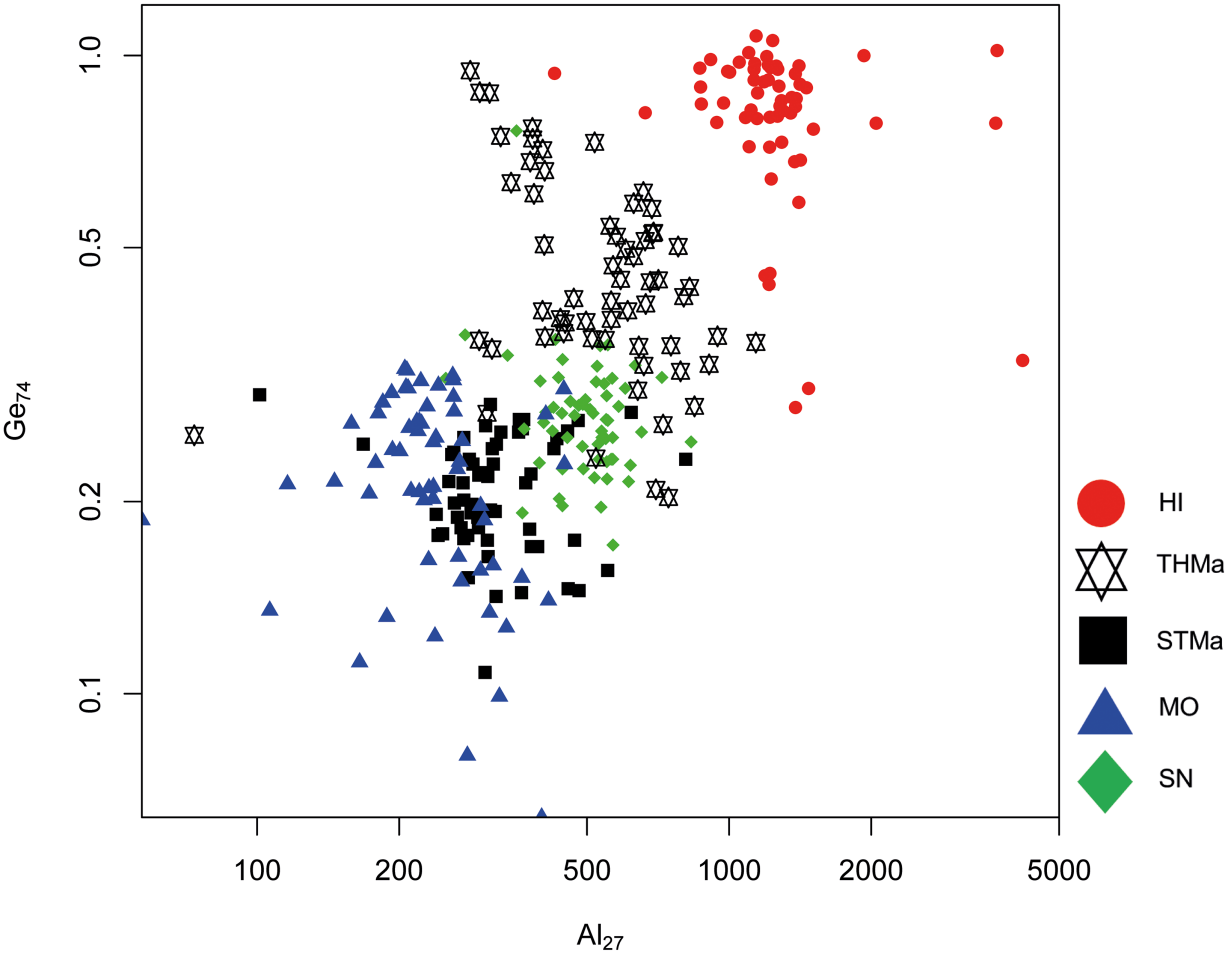
Aluminum (Al) versus germanium (Ge) concentration plot of all Maastrichtian geological samples.

Although overlapping effect occur, trace elements Sr and Ge in combination with Al are suitable to achieve a separation between Maastrichtian sources to a certain degree (Figs 12 and 13). It has to be noted that Ge values are generally very low in Maastrichtian flint samples, ranging between 0.1 and 1 ppm. Therefore, special care was taken to exclude microinclusions and analytical accuracy was closely monitored by repeated measurements of the NIST614 glass reference material which contains Ge in the above mentioned range. However, both Sr–Al and Al–Ge scatter plots allow for a differentiation of three distinct source clusters which are indicative of separate depositional environments. Hence, we were able to identify three different sub-basins within the study area. Elemental concentrations of Sr, Al and Ge display an increasing trend from south to north. Translated into geography, MO, STMa and SN samples fall within the southernmost area, HI is situated in the north, and THMa is located between the two (see Fig 1).

#### CODA results

1. List of variables which are removed, together with the achieved mcCV:

**Table:** 

**Be9**	**Rb85**	**Ti49**	**V51**	**Cr53**	**Fe56**	**Ni60**
0.02000000	0.01666667	0.01666667	0.01666667	0.01666667	0.01666667	0.01666667
**Cu65**	**Mn55**	**Zn66**	**A127**	**Ga71**	**Sr88**	**C059**
0.01666667	0.01666667	0.01666667	0.01666667	0.01666667	0.01666667	0.01666667
**Zr90**	**Y89**	**Cs133**	**Mg24**	**La139**	**Pr141**	**Nd146**
0.01666667	0.01666667	0.01666667	0.01666667	0.01333333	0.01333333	0.01333333
**Gd157**						
0.01333333
2. List of variables that remain for LDA:
  "Li7" "B11" "Ca43" "Ge74" "Ba137" "Ce140" "Sm147" "Eu153" "Dy163" "Er166" "Yb172" "Pb208" "Th232" "U238".
3. LDA with remaining list of variables:
  mcCV = 0.01333333
  Group assignment:

**Table:** 

grp	1	2	3
1	**116**	2	2
2	0	**120**	0
3	0	0	**60**

**Fig 14:**
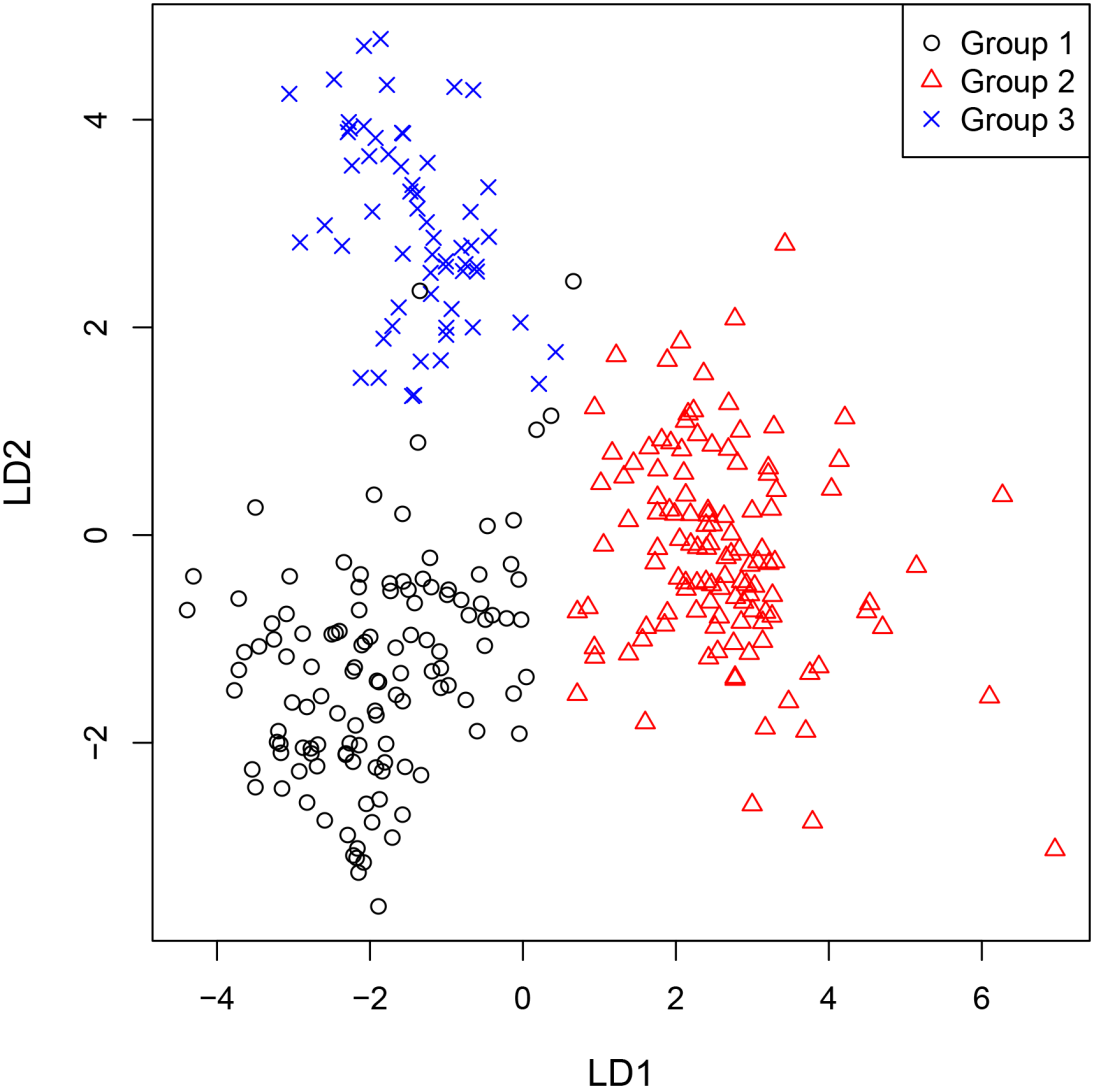
Linear discriminant plot of all Maastrichtian geological samples.

Statistical analyses of the geochemical datasets produced minimal misclassification, and source specific training-data are well allocated as indicated in the assignment table (highlighted numbers). Hence, three distinct source clusters, i.e. depositional basins, were confirmed through CODA application (Fig 14).

#### 3. Danian samples: Bivariate analyses

**Fig 15:**
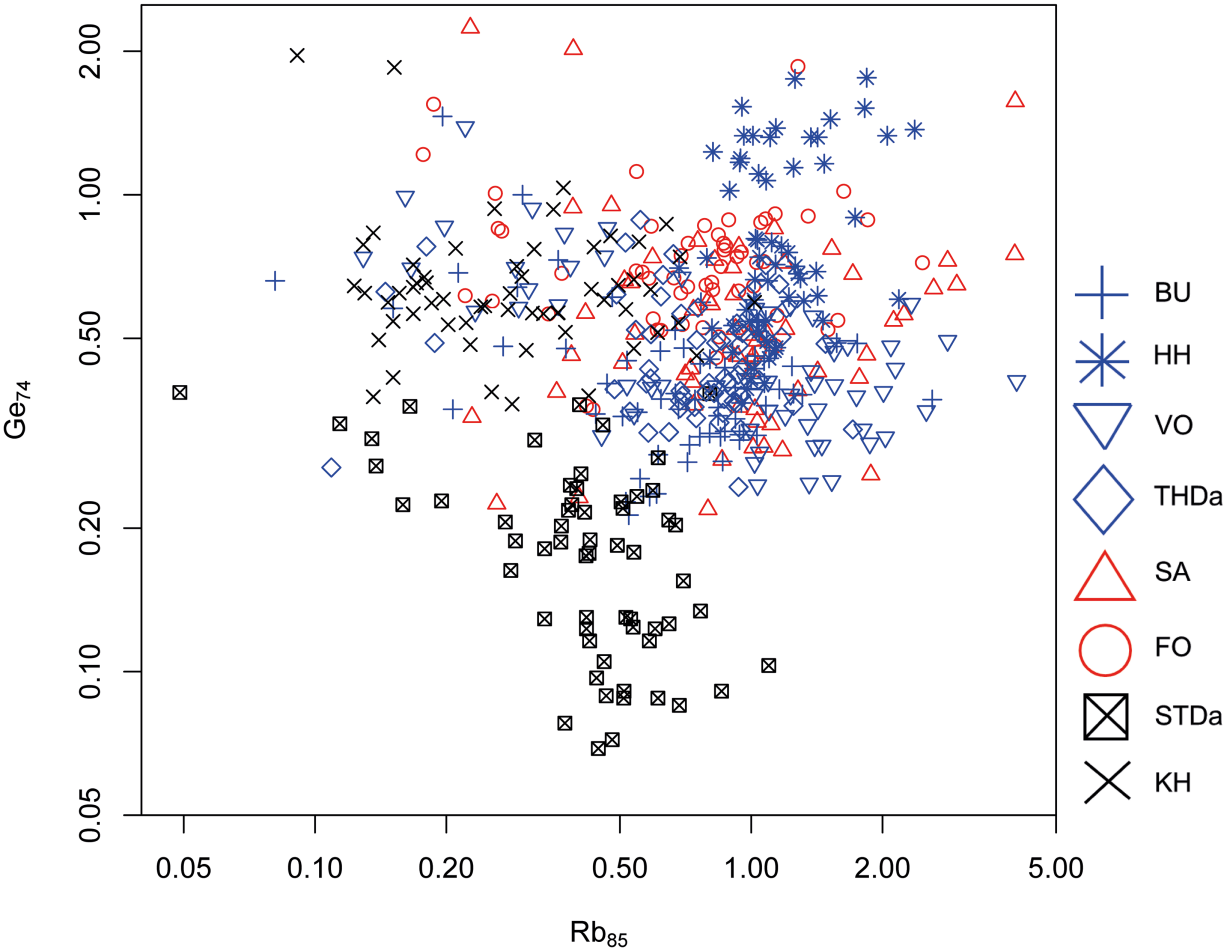
Rubidium (Rb) versus germanium (Ge) concentration plot of all Danian geological samples.

Geochemical differentiation between Danian sources is more intricate. The depositional environments appear far more homogeneous during the Early Paleocene compared to the Maastrichtian. Source separation is only possible to a limited degree. Trace elements rubidium (Rb) and germanium (Ge) provide certain possibilities for geographic discrimination, but overlapping is significant and a clear separation is not possible. Values of both, Rb and Ge, are generally low in Danian flint, with ranges from 0.1 to 5 ppm for Rb, and below 0.1 to approximately 2 ppm for Ge (Fig 15). Only the STDa data cluster is distinct based on extremely low Ge contents, while the rest of the samples scatter insignificantly.

#### CODA results

1. List of variables which are removed, together with the achieved mcCV:

**Table:** 

**Fe56**	**Y89**	**Mn55**	**Th232**	**Gd157**	**Zn66**	**Rb85**
0.12083333	0.11666667	0.11250000	0.10833333	0.10416667	0.10208333	0.10208333
**Ce140**	**La139**	**Ni60**	**Pr141**	**Cs133**		
0.10000000	0.10000000	0.10000000	0.09791667	0.09791667
2. List of variables that remain for LDA:
  "Li7" "Be9" "B11" "Mg24" "Al27" "Ca43" "Ti49" "V51" "Cr53" "Co59" "Cu65" "Ga71" "Ge74" "Sr88" "Zr90" "Ba137" "Nd146" "Sm147" "Eu153" "Dy163" "Er166" "Yb172" "Pb208" "U238".
3. LDA with remaining list of variables:
  mcCV = 0.09791667
  Group assignment:

**Table:** 

grp	1	2	3
1	**223**	9	8
2	18	**100**	2
3	9	1	**110**

**Fig 16:**
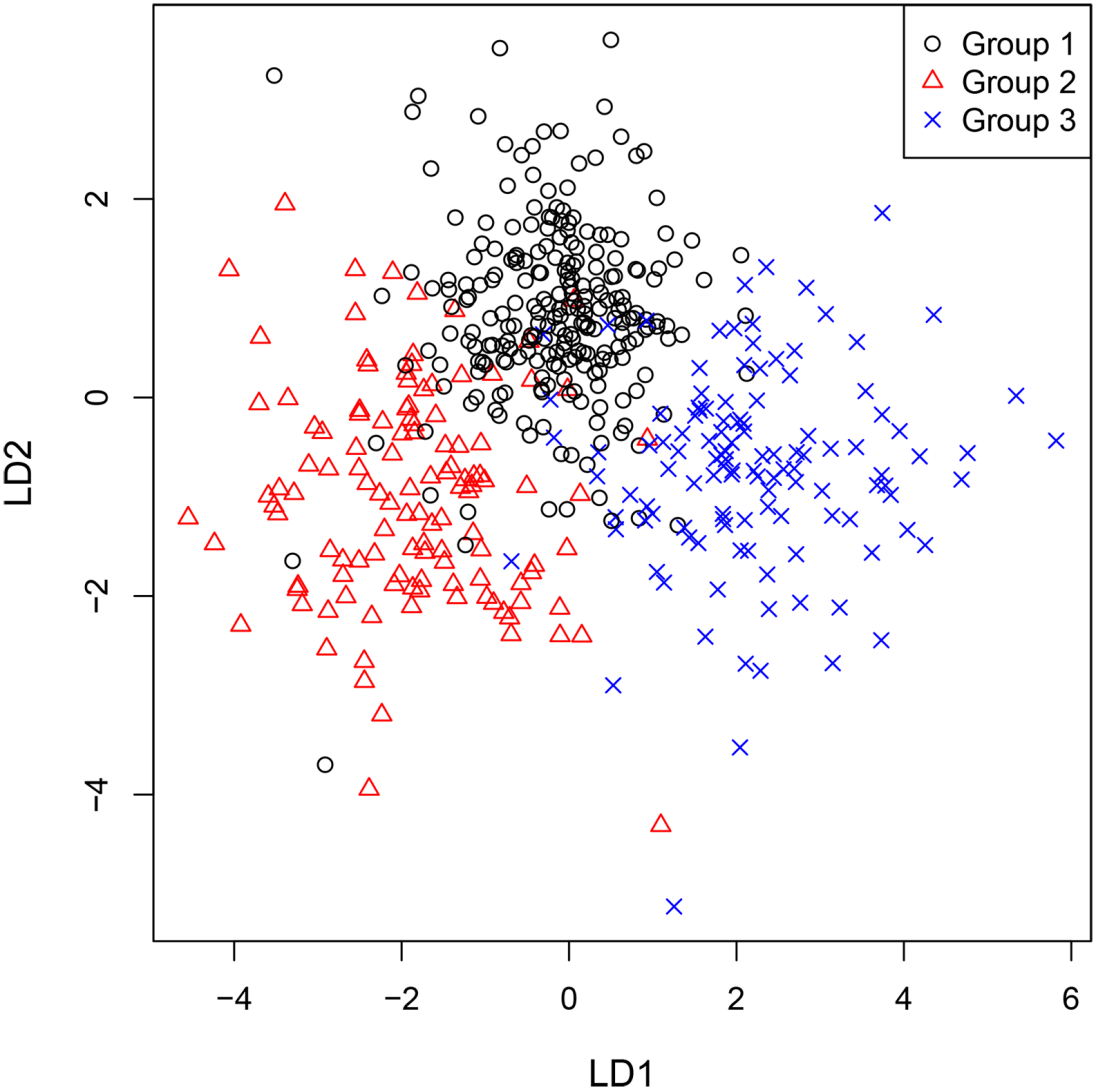
Linear discriminant plot of all Danian geological samples.

A clearer separation can be achieved through statistical analyses. We tested if the Danian succession also allows for internal differentiation and grouping into three discrete source areas. This hypothesis was confirmed using CODA, however, misclassification and therefore overlapping is significantly higher than at Maastrichtian sources (Fig 16).

### Application to the archaeological material

#### Macroscopic (visual) and microscopic analyses

Visually, LBF samples correspond well with Danian limestone with flint inclusions. The flint is porous in its texture, due to abundant calcispheres and micro-skeletal inclusions visible by naked eye. Coloration ranges between dark yellowish brown and almost black with irregularly interspersed light gray patches, displaying an altogether very heterogeneous texture. The investigated material comprises two related flint types consistent with DGB and DBB varieties according to the classification proposed in Table 1. Microscopically, the most apparent feature of LBF is the relative abundance of bryozoan inclusions in the majority of the investigated samples. Abundant Particulate Organic Matter (POM) and peloids are additional characteristics.

#### Microfacies

The texture of all LBF samples corresponds to packed biomicrite, and is identical with geological samples from Danian bryozoan limestone facies.

Macroscopic and microscopic investigations confirm that the LBF material consists of flint bearing Danian bryozoan limestone, which allows defining more specific source criteria. Our field investigations revealed that detached limestone boulders at primary Danian sources are rarely well rounded and considerably larger than the archaeological ship ballast material, since new material is continuously delivered. Danian material which was transported over larger distances and deposited at the shore was in all cases heavily worn and the remaining pebbles too small compared to the LBF sample. Only a deposit located at a short distance from a primary source produces boulders corresponding to the archaeological material. The roundedness of the LBF boulders suggests a source in proximity to the coast, most likely a beach deposit. Additionally, the size of the boulders indicates that they were not transported too far from the primary source. Hence, source conditions for the LBF material match best with a coastal deposit located close by a primary source or an eroded cliff comprised of Danian limestone. Investigated beach sources comprising this kind of material are predominantly located in the north of the study area, however macroscopic assessments are by no means conclusive and need to be substantiated through more refined analyses.

#### Geochemistry

According to the identified source specifics it was reasonable to concentrate on geochemical data obtained from Danian samples for comparison and to assign the LBF to one of the three identified Danian depositional environments.

#### Bivariate analyses

**Fig 17:**
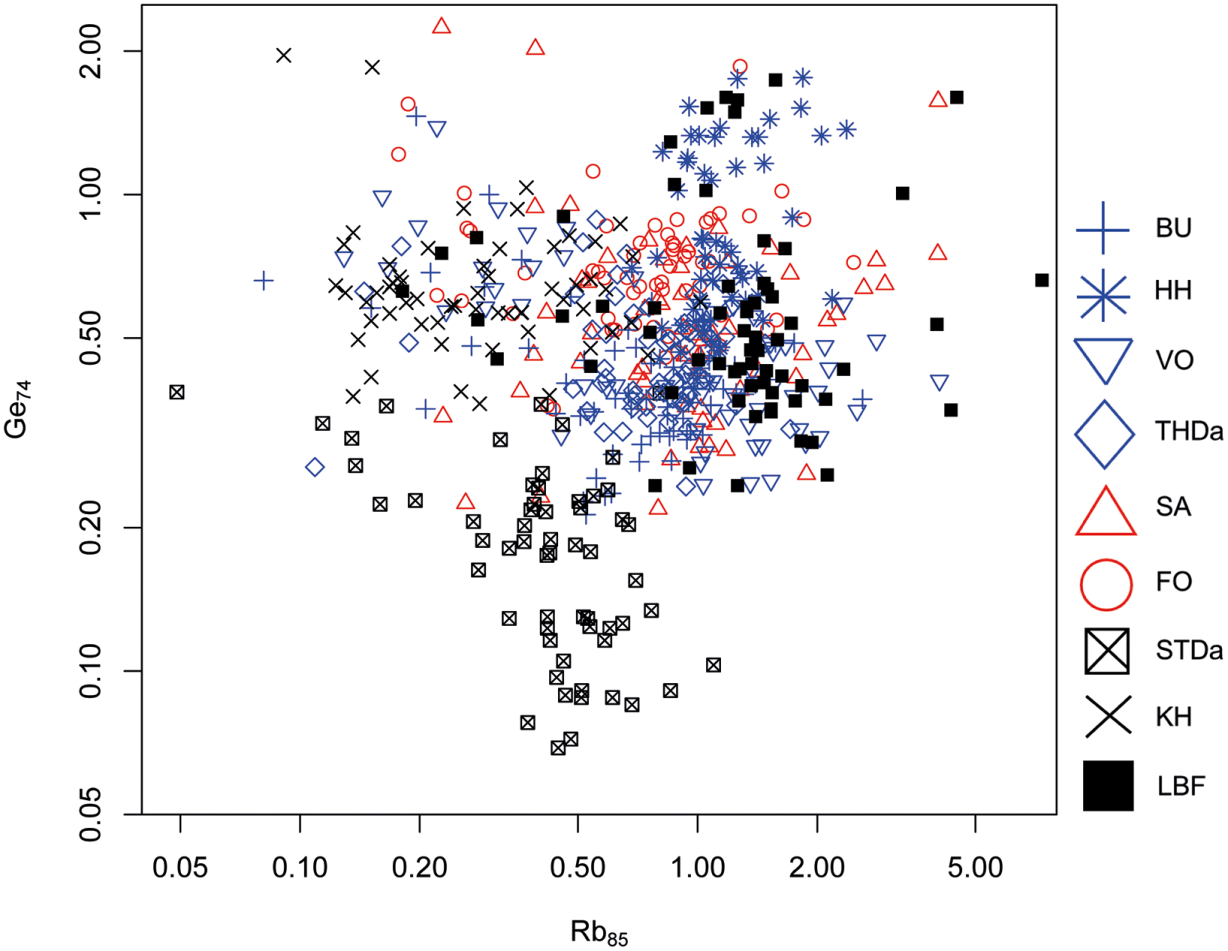
Rubidium (Rb) versus germanium (Ge) concentration plot of the Danian geological samples and the LBF material.

As for the geological samples, trace elements Rb and Ge were used since they allowed for—albeit limited—geospatial resolution between primary Danian sources. Except few outliers, the majority of the LBF data points display a behavior analogous to samples from BU and HH, both situated within the north-westernmost Danian source area (Fig 17). However, since overlapping effects are so significant in the Rb–Ge scatter plot this result could only be considered a mere indication which had to be verified through CODA.

#### CODA results

Prediction of LBF samples using LDA from Danian samples produced the following summarized group assignment:

**Table:** 

Group (training data)	1	2	3
LBF (test data)	**51**	13	2

**Fig 18:**
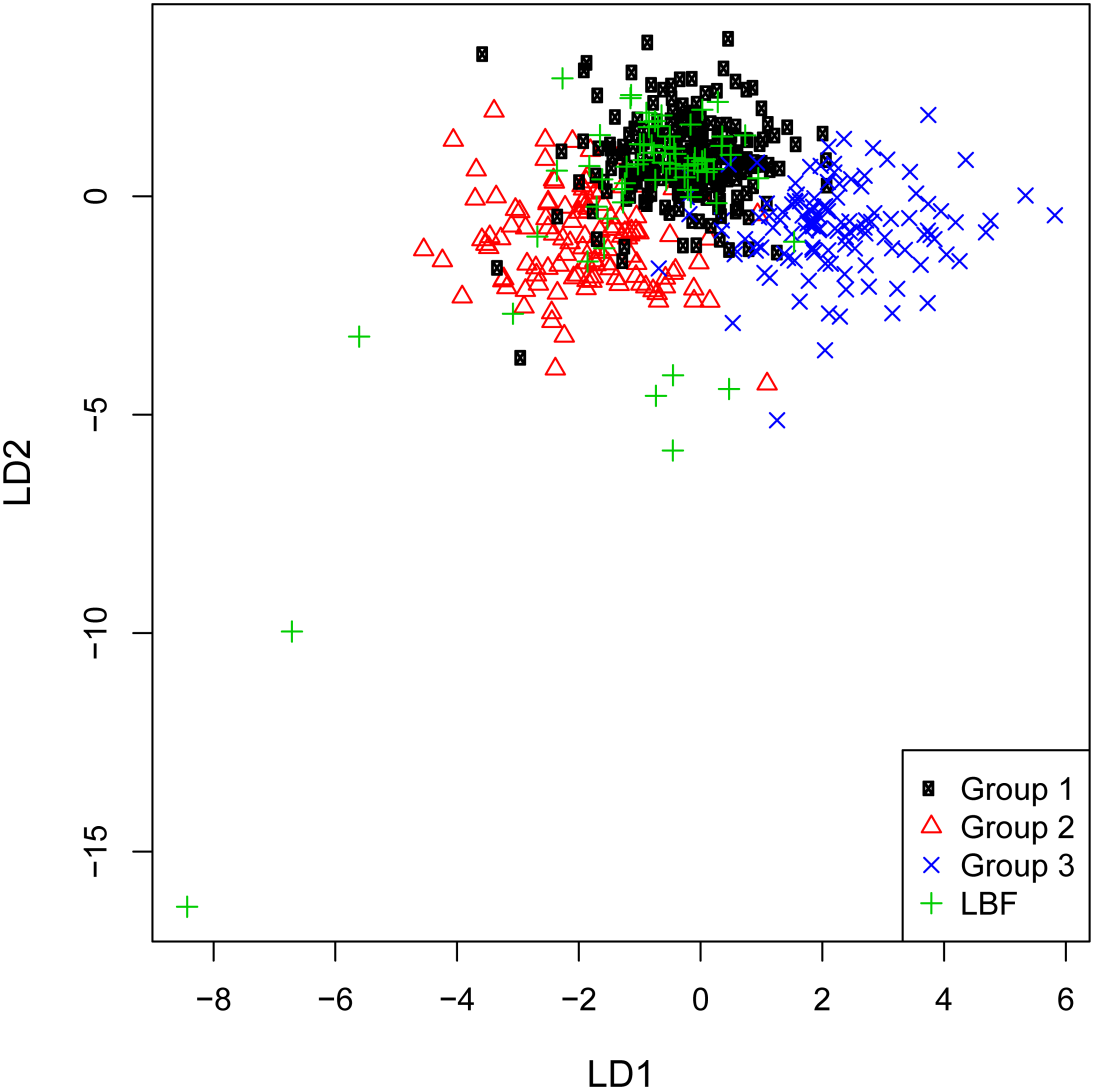
Linear discriminant plot of all Danian geological samples and the LBF material.

As demonstrated by this result and illustrated in Fig 18, the majority of test data (i.e. the archaeological samples) are allocated to group 1 of 3, which were generated from the training data, i.e. geochemical results from Danian flint samples. These three groups correspond to the three geographically distinct Danian source areas. Group 1 of the geological samples represents the northern source cluster within the Danian depositional realm, and the LBF signatures place the archaeological material into this geochemical environment. Hence, the indication derived from Rb–Ge investigations was confirmed.

**Fig 19:**
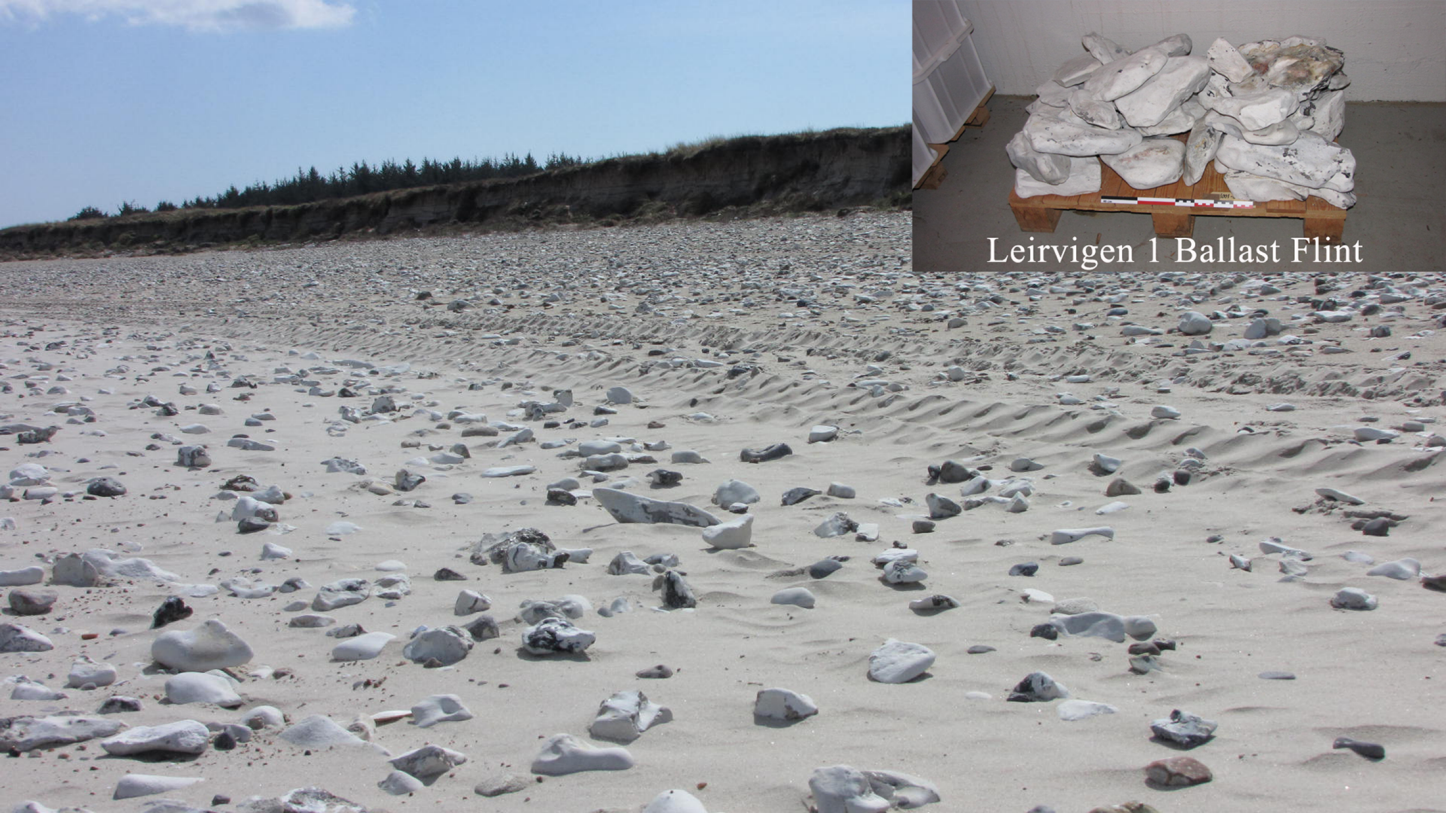
Overview over the Vigsø Bay in Northern Jutland, Denmark.

Macroscopic and microscopic features in combination with geochemical results suggest an origin of the LBF from the northernmost Danian source area, more specifically close to the primary deposits of Hanstholm and Bulbjerg. Our geo-archeological surveys revealed one specific locality which provides material best corresponding with the archaeological finds: A beach stretch between the Bulbjerg (BU) and Hanstholm (HH) sources, the so-called Vigsø Bay. This locale presents an elongated curved beach line characterized by abundant flint bearing limestone boulders exactly corresponding to the LFB material, in terms of size, roundedness, and microscopic properties (Fig 19).

## Discussion

There exist numerous pilot- and also more comprehensive studies attempting to characterize, differentiate and generate a fingerprint for the large variety of micro- to cryptocrystalline siliceous rocks from Northern European chalk and limestone formations generally referred to as “flint” [3] (and further citations therein). However, to date a clear possibility to assign archaeological artifacts to one of the many recognized geological sources has not been achieved due to a higher inter-source homogeneity of certain flint types than intra-source consistency. This problem directly affects what is known as the “Provenance Postulate” stating *“that there exist differences in chemical composition between different natural sources that exceed*, *in some recognizable way*, *the differences observed within a given source”* [86].

Consequently, the main emphasis of the current study was to establish a sound methodology for sourcing Scandinavian flint based on hypotheses that were formulated after a decade of provenance studies involving silicites from a broad variety of geological backgrounds [69, 70, 73, 87, 88]. Detailed geological and morphological information is available for each geological location discussed in this study, hence we concentrated on parameters we found best suitable for source differentiation. The hypotheses we tested through this investigation are of geological nature. Many provenance studies attempt to differentiate samples from a geological sequence or profile which in most cases does not produce significant results [3, 4]. Potentially such an approach can be useful on a micro-scale, however results can be misleading when a differentiation between deposits on a larger scale is endeavored. The explanation can be found in the nature of the deposits, directly linked to the genetic environments of the analyzed materials. As previous studies have revealed, two significant trends for chert source provenance studies applying geochemistry are evident. The first trend is spatial, the second one chronological.

In marine environments, chemical variability is recognizable between basin structures which may be spatially extensive. In such submarine basins or troughs, trace element concentrations of the sea water display fairly homogeneous distributions reflected in trace element contents. Intra-basin differences of the values are due to different depositional regimes under which silicites were formed. This is directly linked to distinct locations within the basins, i.e., ridge-proximal (mid-ocean ridges), pelagic (open ocean), and at continental margin environments. While the sedimentation rate is considerately higher in silicites formed at continental margins due to continental input, silicites bound to ridges or developed under pelagic conditions were exposed to the seawater for longer time periods, which is reflected in REE and trace element deposition [5, 89, 90].

Trace elements strontium (Sr), aluminum (Al), titanium (Ti), magnesium (Mg), manganese (Mn), germanium (Ge) and rubidium (Rb) were found to be suitable for chemically sourcing Scandinavian flint. Primary sources of Sr, Al, Ti, Mg, Mn, Ge and Rb in seawater are terrigene input from weathering of a wide variety of minerals and hydrothermal fluids. In sea sediments, Al is indicative of feldspars, mica or clay minerals, Rb is present mainly in K-feldspar, mica and clay minerals, and Mg typically indicates dolomite. Manganese content is an important indicator for degradation of organic matter [91–94]. Germanium is strongly correlated with silicon (Si) in the marine cycle, suggesting that the distribution of both elements is controlled by the same mechanisms. Accordingly, Ge content can be used to reconstruct changes of the Si concentration in the past deep sea [95]. Especially this set of trace elements is absorbed and accumulated by microorganisms and eventually into biogenic opal, and can thus be considered proxies indicative of changes in bioproductivity. As demonstrated by the current study, such elements can allow a differentiation between distinct source areas to various degrees, and in some cases between sources within a considerably small catchment area, e.g. depositional basins. However, such intra-basinal differences were found to be less significant than inter-basinal signatures in their trace element composition.

Accordingly, differentiation is in most cases possible between sources linked to different depositional regimes (frequently separated by basin structures), whereas outcrops situated within the same genetic environment can often not be clearly separated geochemically [70]. A finer resolution can be achieved through microfacies analysis, revealing the depositional environment of silicites located at the neritic, benthic or pelagic zone [44, 96].

The second issue is chronological. Since the chemical signatures of geological profiles displaying multiple and oftentimes closely stacked silicite layers produce ambiguous results, a secure differentiation is only possible between layers which are separated by a change in the chemistry of the host rock environment. This may be caused by environmental changes, altered sedimentation processes, increased or decreased terrigene influx (e.g. through rivers), or a combination of some of these factors. Hence, only silicites derived from distinct host rock facies (regardless if they derive from one outcrop or distant sources) can be securely distinguished, as demonstrated by our results.

### Ship ballast

Sourcing flint used as ship ballast is a promising approach to further explore economic aspects of this archaeological marker. Ballast can be used to localize harbor sites, more specifically ports of call, and understand harbor management strategies such as the procurement, use, recycling and final deposition of materials used as ship ballast and maritime trade networks [97–110].

Successfully sourcing ballast of the Leirvigen 1 shipwreck to the Vigsø Bay raises the main economic question concerning why and how this specific material was used. Source location and material type suggest two possibilities for an interpretation of the archaeological record, both of which are equally possible:

The ballast boulders were directly gathered from the beach at Vigsø at which the ship had one of its stops. The flint boulders may have been taken on board

1. in the course of its initial loading of this particular journey, or
2. during one of the stopovers.

It is to date not possible to decide which alternative is more likely. However, the ship can be placed into an economic scenery of the time between 1450 and 1650 as indicated by an existing C^14^ date from one of the ship`s planks. This date and the geographical location of the shipwreck indicate a possible connection with the so-called “Skudehandel” for timber trade between Denmark and Norway between the late 15th and early 19th centuries [111–113].

## Conclusion

Translating Weigand`s Provenance Postulate [86] referred to at the beginning of the discussion into a multi-methodological approach such as the MLA, successful silicite provenance studies depend on the systematic combination of analytical procedures revealing recognizable differences in visual characteristics, source specific microscopic features, and chemical composition between geographically separated natural sources exceeding heterogeneities observed within a given source. The careful compliance to this approach allows tracing lithic artifacts back to their original source area as demonstrated by this study. However, successful implementation is highly contingent on the scientific questions: Failure of analytical procedures for provenance studies is oftentimes a result of inadequate questions to the studied materials. For the current study, we had specific hypotheses which were tested through the application of the MLA chert sourcing technique. Our results derived from systematic analytical work demonstrate clear possibilities for distinguishing materials from primary Scandinavian flint deposits on different scales, and it was possible to establish the origin of the archaeological material.

For Scandinavian flint, we were able to demonstrate that combined visual and microscopic analyses only permit a differentiation between Maastrichtian and Danian deposits with sufficient certainty. To achieve a finer resolution, geochemistry—here LA-ICP-MS analytics—coupled with CODA for statistical evaluation of geochemical data was employed. In an initial step, we investigated the potential to differentiate sources of Late Cretaceous (Maastrichtian) and Early Paleocene (Danian) age throughout the study region. Subsequently, we tested possibilities to group and distinguish source clusters within those depositional regimes, which ultimately revealed chemically distinct source areas indicative of individual depositional basins. Once this was achieved, it was possible to assign any particular source to one of the defined source areas. Including the archaeological material, analytical results suggest that the Danian limestone boulders used as ballast on the Leirvigen 1 ship originate from a source in the north of the study area (i.e. Denmark), situated close to a primary outcrop on the shore. Visual and microscopic characteristics in combination with geochemical and statistical results reveal the area around Hanstholm and Bulbjerg as the most likely source region of the LBF material. The nature of beach deposits recorded during systematic field surveys of the entire Danish and north German secondary flint-bearing shore zone additionally supports this assessment. Within the study region, there exists only one distinct area which fulfills all requirements for the LBF: the Vigsø Bay in Northern Jutland. Primary sources for the boulders at Vigsø Bay are located to the north at Bulbjerg and possibly in the south around Hanstholm. Hence, we conclude that the most likely source of the LBF can be located at this particular part of the Danish coast. Considering the location of the shipwreck as indicated in Fig 1, this conclusion is particularly convincing, and demonstrates the value of our systematic methodological approach.

The outcomes of the present study offer potential for sourcing flint implements from various archaeological contexts on a pan-European scale. In the future, additional primary deposits will be included into our database to test and consolidate our geological findings, and more archaeological material needs to be investigated.

## Supporting information

S1 Fig Macroscopic and microscopic comparison of all geological samples and the Leirvigen 1 ballast flint material. (TIF)

S1 Table LA-ICP-MS data. The table contains the elemental concentration values of samples from Maastrichtian and Danian flint sources. Maastrichtian samples derive from Hillerslev (HI), Thisted (THMa), Stevns (STMa), Møn (MO) in Denmark and Sassnitz (SN) on Rügen in Germany. Danian sources are represented by Bulbjerg (BU), Hanstholm (HH), Vokslev (VO), Thisted (THDa), Sangstrup (SA), Fornæs (FO), Stevns (STDa) and Klintholm (KH). Ballast Flint samples from the Kristiansand Leirvigen 1 shipwreck are indicated as LBF samples. Only samples suitable for LA-ICP-MS analysis were included in this study, hence sample numbers are random. Three separate spots were analyzed for each geological sample. Archaeological material was sometimes analyzed on six spots for control reasons. All values are provided in parts per million (ppm). The data have not been logarithmically adjusted.
(XLSX)

S2 Table Individual feature description of all investigated samples including the LBF material. (XLSX)
