# Supplementary figures and images for: A multi-technique analytical approach to sourcing Scandinavian flint: Provenance of ballast flint from the shipwreck “Leirvigen 1”, Norway
Source: PLoS One. 2018 Aug 8;13(8):e0200647. doi: 10.1371/journal.pone.0200647 (PMC6082525; doi:10.1371/journal.pone.0200647)

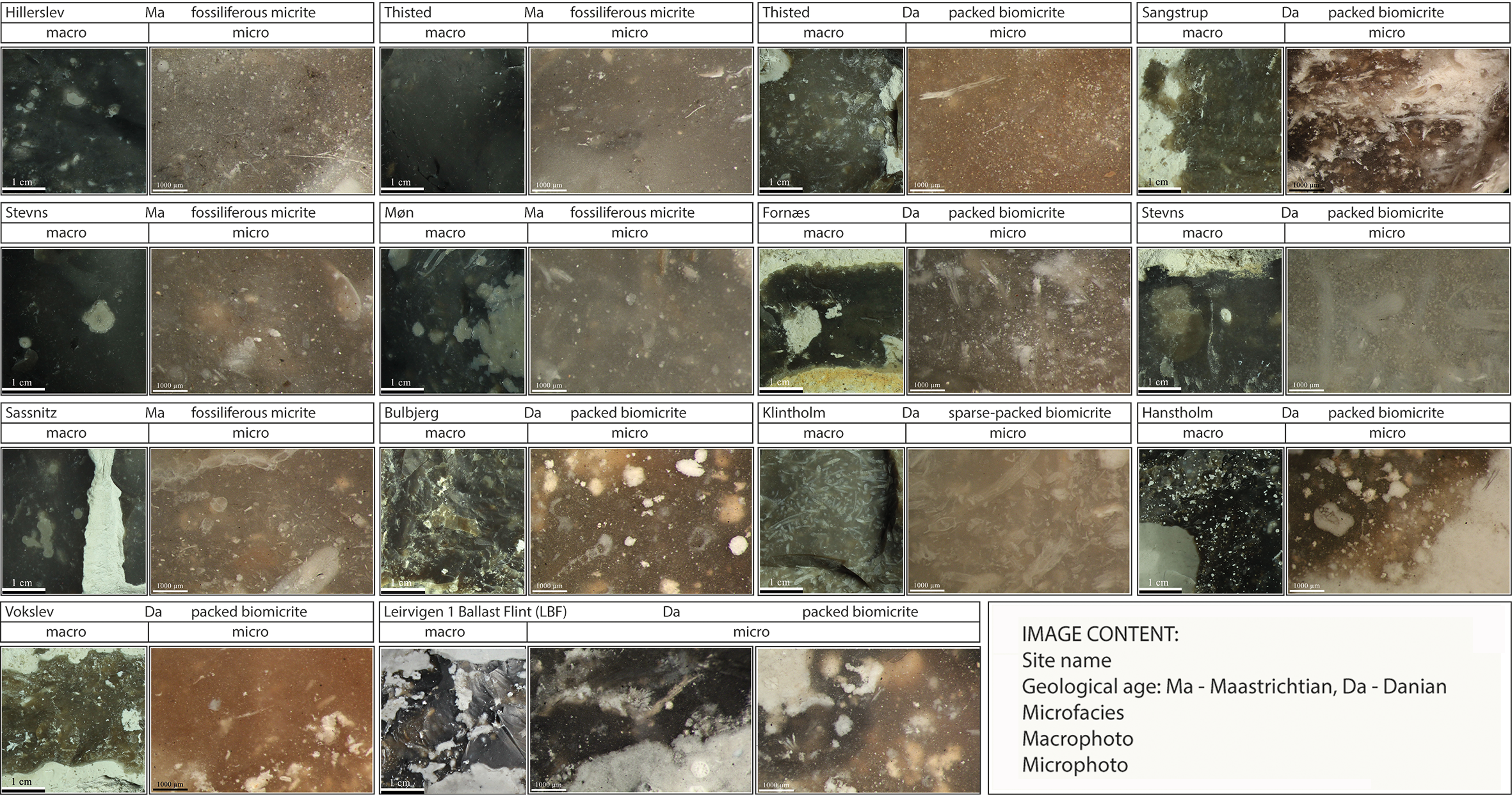

Supplement: S1 Fig — (TIF) [file pone.0200647.s001.tif]
